# Magnetic-Field-Assisted CO_2_ Electroreduction at Precision-Engineered Ga–Gd Oxide Nanodomain Interfaces

**DOI:** 10.1021/prechem.5c00435

**Published:** 2026-02-11

**Authors:** Mohammad Karbalaei Akbari, Kumar Shrestha, Noor Aljammal, Alireza Pourvahabi Anbari, Yanbin Cui, Serge Zhuiykov

**Affiliations:** † Department of Solid-State Sciences, Faculty of Science, 26656 Ghent University, Krijgslaan 281/S1, B-9000 Ghent, Belgium; ‡ Center for Green Chemistry & Environmental Biotechnology, Ghent University Global Campus, Incheon 21985, South Korea; § Laboratory for Chemical Technology (LCT), Department of Materials, Textiles, and Chemical Engineering, 26656 Ghent University, Technologiepark 125, 9052 Ghent, Belgium; ∥ Department of Chemistry, Faculty of Science, 26656 Ghent University, Ghent B-9000, Belgium; ⊥ Institute of Process Engineering, Chinese Academy of Sciences, Beijing 100190, China

**Keywords:** magnetic oxide nanodomains, multiphase oxide nanocomposites, magnetic field-assisted catalysis, CO_2_ electroreduction, spectroscopic characterization

## Abstract

Liquid-metal-derived catalysts offer a unique platform for precision chemistry by enabling structurally adaptive interfaces unconstrained by rigid lattices. Here, we report a magnetically responsive Ga–Gd catalytic system in which ultrafine, surface-localized Gd-rich oxide nanodomains form within a liquid-metal-derived Ga_2_O_3_ matrix and enable field-sensitive electrochemical CO_2_ conversion. Controlled thermal annealing transforms disordered Ga–Gd composites into a defect-suppressed β-Ga_2_O_3_ framework decorated with nanometric Gd_2_O_3_ domains, establishing localized electronic and paramagnetic environments without bulk lattice substitution. Atomic-resolution microscopy, XRD, Raman spectroscopy, XPS/UPS, and solid- and liquid-state NMR reveal annealing-induced suppression of defect states, relaxation of surface dipoles, and emergence of localized magnetic interactions at the Ga–Gd–O interface. Under electrochemical CO_2_ reduction, annealed Ga–Gd electrodes exhibit pronounced magnetic modulation of activity, with CO_2_ conversion increasing from ∼10% at zero field to ∼14–15% under a 200 mT magnetic field (enhancement factor ∼ 1.4–1.5). Product-resolved spectroscopy confirms selective formation of CO (up to ∼0.42 mmol g^–1^ h^–1^) and CH_3_OH (up to ∼0.18 mmol g^–1^ h^–1^) while excluding hydrocarbon and C–C coupling pathways. The magnetic enhancement is potential-selective and confined to the CO_2_ activation window, indicating field-sensitive interfacial kinetics rather than bulk transport effects. These results highlight how ultrafine oxide nanodomains within liquid-metal-derived catalysts can serve as precision-defined, reconfigurable interfacial motifs whose electrochemical reactivity can be dynamically modulated by external magnetic fields, advancing controllable CO_2_ conversion within the framework of precision chemistry.

## Graphical abstract

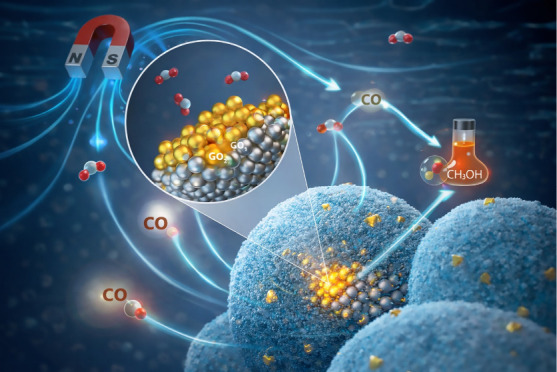

## 1 Introduction

Single-atom catalysis has emerged as an important framework for maximizing atom efficiency and achieving well-defined active sites through precise control of local coordination environments.^1,2^ By isolating catalytic centers at the atomic scale, single-atom catalysts (SACs) enable detailed interrogation of structure–activity relationships and have demonstrated impressive selectivity in a range of electrochemical reactions. Despite these advances, a fundamental limitation remains: in most SAC systems, the atomic coordination environment is structurally fixed by the host lattice or support.^3,4^ As a result, catalytic selectivity and kinetics are largely determined at the point of synthesis and cannot be dynamically adjusted during operation. This constraint is particularly restrictive for electrochemical CO_2_ reduction (CO_2_RR), where subtle changes in interfacial charge distribution, electronic structure, and local coordination can strongly influence reaction pathways and product distributions.^5,6^ While SACs have achieved high selectivity toward CO and other C_1_ products, externally addressable and reversible modulation of single-atom–like active sites during electrochemical operation remains an outstanding challenge.^7^

Addressing this challenge requires catalytic platforms that combine atomic-scale precision with structural adaptability. In this context, gallium-based liquid metals, and Galinstan (Ga–In–Sn) in particular, offer a fundamentally different materials paradigm.^8^ Unlike rigid crystalline supports, gallium-based systems exhibit weak lattice constraints, high atomic mobility, and pronounced interfacial heterogeneity.^9,10^ Even when processed into nanoparticulate or oxide-stabilized forms, gallium-rich materials retain an unusual capacity for interfacial reorganization in response to external stimuli.^11,12^ These properties make gallium-based materials attractive hosts for single-atom–like or few-atom catalytic motifs whose coordination environment is not permanently locked, but instead stabilized within a chemically adaptive matrix.^13^ A key feature of Galinstan is its tendency toward galvanic replacement and surface-mediated metal incorporation, enabling foreign metal species to be introduced as spatially localized atomic or subnanometric domains rather than extended bulk alloys.^14,15^ Such domains can resemble single-atom catalytic sites in terms of coordination and electronic isolation, while remaining embedded in a dynamically responsive Ga–In–O environment.

In parallel, magnetic-field-assisted catalysis has gained renewed attention as a means to modulate electron transport, spin polarization, and interfacial kinetics without altering catalyst composition.^16–18^ For magnetic fields to influence catalytic behavior beyond secondary transport effects, two conditions must be satisfied: the presence of magnetically active atomic species and a host environment that allows those species to respond electronically or structurally to the applied field.^19–21^ In rigid solid supports, these requirements are rarely met simultaneously, and magnetic effects are often limited to magnetohydrodynamic phenomena.^22,23^ A recent study on iron-incorporated gallium-based liquid metal catalysts provided a notable exception, demonstrating reversible magnetic-field-induced reconfiguration between single-atom and clustered states accompanied by changes in reaction pathways.^24^ Importantly, this work established that magnetic fields do not directly alter molecular adsorption energies, but instead act as external regulators of atomic configuration, spin alignment, and interfacial electronic structure. This insight provides a conceptual bridge between magnetic-field modulation and precision single-atom chemistry. Building on this framework, we report here a Galinstan-derived Ga–Gd oxide nanodomain catalyst designed to enable magnetic-field-assisted CO_2_ electroreduction through controlled atomic-scale interfacial states. Gadolinium is particularly well suited for this role due to its large magnetic moment, strong spin–orbit coupling, and ability to form stable oxide-coordinated environments.^25,26^ When incorporated at low loading within a gallium oxide matrix, Gd can exist in few-atom coordination motifs, providing magnetically addressable interfacial control points.^27,28^

The synthesis strategy and working principle are summarized schematically in Figure 1. Galinstan nanoparticles are first produced by ultrasonication in anhydrous ethanol, yielding spherical nanoparticles that preserve the multicomponent Ga–In–Sn composition (Figure 1a). Introduction of ∼5 nm gadolinium nanoparticles during sonication triggers a galvanic interaction at the Galinstan surface. Rather than forming a homogeneous alloy, Gd becomes preferentially localized at the nanoparticle surface, generating discrete nanoscale domains composed of atomically dispersed or subnanometric Gd species (Figure 1a,b), consistent with prior reports on Galinstan-mediated metal decoration chemistry.^28^ Subsequent annealing under argon with controlled oxygen exposure induces partial oxidation of the gallium-rich surface, forming a Ga–In–O matrix that stabilizes the nanoparticle architecture while anchoring Gd in low-coordination oxide environments. Structural studies confirm the formation of Gd- and Gd_2_O_3_-rich surface nanodomains rather than uniformly doped oxides or encapsulated magnetic particles, preserving surface-accessible, well-defined Gd sites. From a precision-chemistry perspective, the Ga–In–O matrix functions as a chemically adaptive support that stabilizes single-atom-like Gd motifs while permitting their electronic and spin states to respond to an external magnetic field. As illustrated in Figure 1c, these Gd-rich regions act as interfacial modulation sites for CO_2_-derived intermediates by influencing local charge density, spin polarization, and electron-transfer kinetics at the oxide-electrolyte interface. Under electrochemical CO_2_ reduction conditions (Figure 1d,e), the application of a magnetic field biases the electronic and spin configuration of Gd-centered sites without introducing new thermodynamic pathways. Instead, magnetic modulation reshapes reaction kinetics and intermediate populations, manifesting experimentally as changes in electrochemical response and product signal intensities.^29,30^ ^,^ ^31^ In this work, we therefore position the Galinstan-Gd system as a precision-engineered, field-responsive catalytic interface that extends few-atom catalysis concepts beyond static active sites. By integrating detailed structural characterization with electrochemical analysis and spectroscopic product verification (Raman, FTIR, and NMR), we establish a coherent link between atomic-scale interfacial structure, magnetic-field-induced modulation, and CO_2_-to-CO conversion behavior. This approach demonstrates how precision chemistry principles can be applied to dynamically reconfigurable atomic environments, offering new opportunities for externally controlled electrochemical transformations.

**Figure 1:**
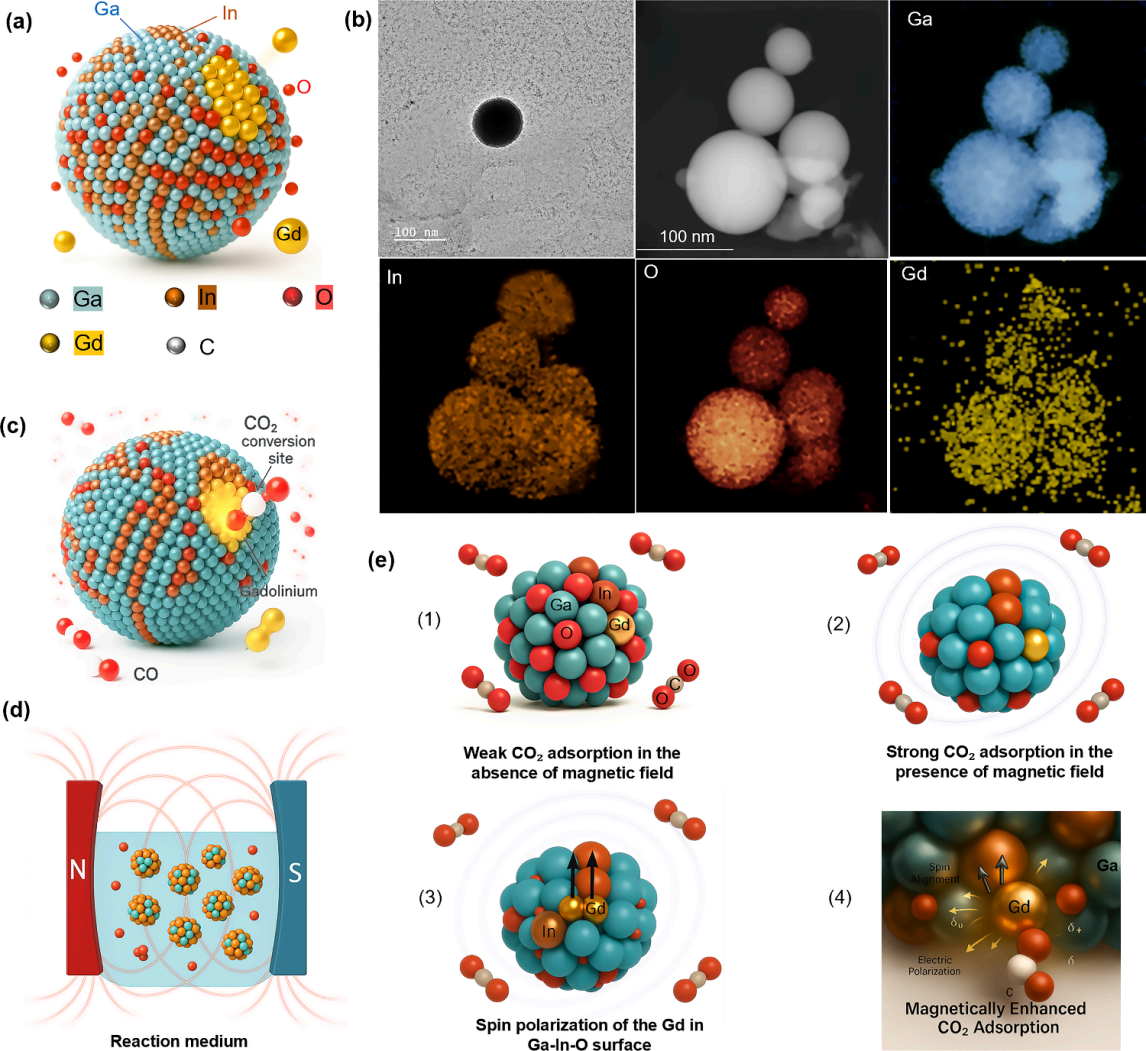
Conceptual schematic of magnetically modulated CO_2_ electroreduction on Ga–Gd oxide nanocomposites. (a) Ga–In–O nanocomposite incorporating ultrafine Gd-containing nanodomains. (b) Representative microscopy and elemental maps showing particle morphology and spatially dispersed Ga, In, O, and Gd phases. (c) Localized CO_2_ adsorption and activation at Gd-enriched surface sites. (d) Reaction environment under an external magnetic field. (e) Proposed mechanism in which magnetic-field-induced spin polarization and local electronic perturbation at Gd sites enhance CO_2_ adsorption and interfacial charge transfer: (e-1) baseline CO_2_ interaction with the Ga–In–O surface depicting the weak CO_2_ adsorption in the absence of magnetic field, (e-2) strong CO_2_ adsorption in the presence of magnetic field, (e-3) spin polarization of Gd centers at the Ga–In–O interface in the presence of magnetic field, and (e-4) magnetically enhanced CO_2_ adsorption and activation.

## 2 Results and Discussion

### 2.1 Structural Transformation of Galinstan-Gd Nanoparticles

Figure 2 summarizes the structural outcome of the Galinstan-Gd nanopowder synthesis after thermal annealing and establishes the baseline crystallographic state of the material prior to heterointerface analysis (Figure 3). At this stage, the emphasis is on identifying the dominant phases, crystallinity, and oxidation pathway derived from the liquid-metal precursor, rather than resolving atomically specific Ga–Gd interfacial motifs. Low-magnification TEM images of the annealed nanoparticles (Figure 2a) show compact, near-spherical particles with characteristic diameters of several tens of nanometers. The preservation of particle integrity after annealing is notable, as uncontrolled oxidation of liquid gallium systems often results in shell rupture, void formation, or fragmentation due to volumetric expansion and stress accumulation. The absence of such features indicates that oxidation proceeds gradually and coherently, consistent with an inward-growing oxide front rather than abrupt surface passivation.

**Figure 2:**
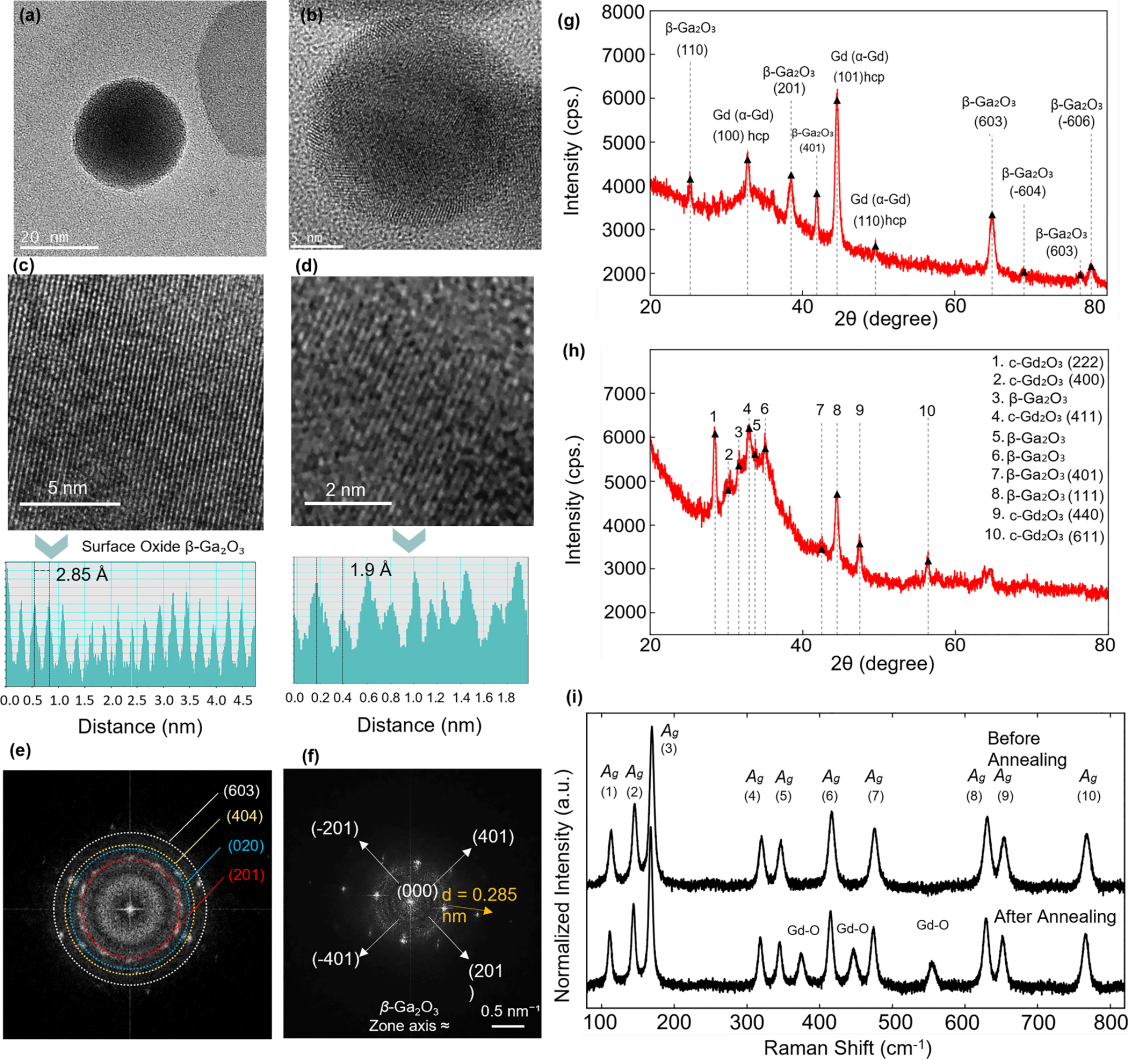
Structural and phase evolution of Ga–Gd oxide nanocomposites upon annealing. (a,b) TEM and HRTEM images showing spherical nanoparticles with nanocrystalline interiors and exterior. (c,d) Lattice-resolved HRTEM revealing distinct crystalline domains with their corresponding interlayer-distancing graphs measured *d*-spacings at the bottom of each crystalline HRTEM image. (e,f) FFT and indexed diffraction patterns confirming β-Ga_2_O_3_ crystallinity. (g,h) XRD patterns before and after annealing evidencing phase refinement and emergence of c-Gd_2_O_3_ alongside β-Ga_2_O_3_. (i) Raman spectra showing stabilized Ga–O and Gd–O vibrational modes after annealing, consistent with improved oxide ordering.

**Figure 3:**
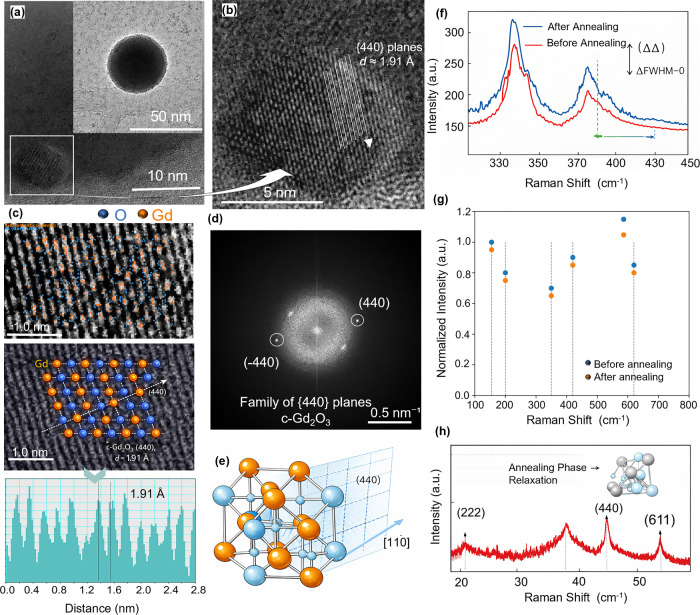
Annealing-driven structural ordering of Ga–Gd oxide nanodomains. (a,b) HRTEM images of annealed nanoparticles showing the emergence of coherent lattice fringes. (c) Atomically resolved contrast and schematic overlay illustrating Gd-enriched nanodomains embedded within the Ga–In–O host lattice. (d) FFT pattern indexed to the {440} family of β-Ga_2_O_3_, confirming host-lattice crystallinity and nanoscale ordering. (e) Structural model illustrating the orientation of Gd-containing oxide nanodomains relative to the β-Ga_2_O_3_ lattice. (f,g) Micro-Raman spectra and normalized peak-intensity analysis showing phonon sharpening and symmetry evolution upon annealing, consistent with reduced structural disorder. (h) Local diffraction/XRD evidence indicating short-range ordering of Gd-containing oxide domains after annealing, complementing bulk XRD results.

High-resolution TEM imaging (Figure 2b) reveals lattice fringes extending across substantial regions of individual particles, indicating that thermal annealing promotes the formation of extended crystalline domains rather than a thin amorphous Ga–O layer. This behavior is consistent with previous studies showing that controlled oxidation of liquid gallium favors crystalline β-Ga_2_O_3_ over disordered gallium oxyhydroxides. Atomic-resolution HRTEM of the outer particle region (Figure 2c) resolves a well-defined interplanar spacing of ∼2.58 Å, extracted from line-profile analysis, closely matching reported lattice spacings of monoclinic β-Ga_2_O_3_.^32,33^ The presence of this spacing at the particle periphery confirms that the surface oxide is crystalline and phase-selective, and that gadolinium incorporation does not suppress β- Ga_2_O_3_ nucleation. In addition to this dominant spacing, a second lattice motif with an interplanar distance of ∼1.9 Å is observed in the IFFT-reconstructed HRTEM image in Figure 2d. This spacing overlaps with reported values for higher-index β-Ga_2_O_3_ planes^32,33^ as well as cubic c-Gd_2_O_3_ reflections.^34,35^ Rather than assigning this feature to a single phase, its coexistence with the 2.58 Å lattice within the same particle indicates local heterogeneity in oxide coordination environments, suggesting that gadolinium participates in oxide formation in a manner that introduces additional crystallographic motifs^36^ beyond pure β-Ga_2_O_3_. Selected-area electron diffraction (SAED, Figure 2e) further bridges atomic-scale imaging and ensemble crystallography, displaying concentric diffraction rings predominantly indexed to β-Ga_2_O_3_, including (−201), (020), (404), and (603) reflections.^37,38^ The ring-like pattern confirms a nanocrystalline, polyoriented structure with limited amorphous content.

FFT patterns extracted from high-resolution regions (Figures 2f and S1) show discrete diffraction spots indexed to monoclinic β-Ga_2_O_3_ along a zone axis close to [010], with clearly resolved (201), (−201), (401), and (−401) reflections and a characteristic interplanar spacing of ∼0.285 nm. These locally ordered regions demonstrate that annealing produces nanometer-scale domains approaching single-crystal order,^39^ despite the overall nanocrystalline nature of the material, enabling well-defined atomic coordination environments to emerge from the liquid-metal-derived precursor. The coexistence of the 2.58 Å β-Ga_2_O_3_ lattice (Figure 2c) with the 1.9 Å motif (Figure 2d), together with the [010]-oriented β-Ga_2_O_3_ FFT in Figure 2f, confirms that gadolinium incorporation introduces locally modified oxide coordination environments within an overall β-Ga_2_O_3_ crystallographic framework rather than forming a separate bulk Gd_2_O_3_ phase.

These microscopy observations are directly corroborated by X-ray diffraction. Before annealing (Figure 2g), the diffraction pattern contains contributions from residual metallic In/Sn phases alongside weak, broadened oxide reflections, indicating incomplete oxidation and substantial lattice strain. After annealing (Figure 2h), sharp reflections associated with β-Ga_2_O_3_ dominate the pattern, accompanied by additional peaks attributable to Gd-containing oxide phases, including c-Gd_2_O_3_ and mixed Ga–Gd–O signatures, while metallic Galinstan peaks persist at reduced intensity. Quantitative phase analysis and peak assignments are provided in the Supporting Information Note 1, Tables S1–S3. Williamson–Hall analysis^40^ further quantifies this structural evolution, revealing that the β-Ga_2_O_3_ microstrain decreases from approximately 10^–3^ before annealing to ∼10^–4^ after annealing, while the crystallite sizes converge to a stable range of 18–22 nm for both β-Ga_2_O_3_ and c-Gd_2_O_3_ phases (Figure S2 and Table S4). This pronounced reduction in microstrain indicates substantial lattice relaxation during annealing, accompanied by redistribution and partial annihilation of strain-related defects introduced during liquid-metal oxidation. Importantly, the stabilization of crystallite size alongside strain reduction suggests that annealing does not induce uncontrolled grain growth, but instead promotes structural equilibration at the nanoscale. Such relaxation is consistent with the emergence of sharper diffraction and vibrational features and implies the formation of more uniform and well-defined oxide coordination environments, a prerequisite for stable atomic-scale active sites in precision catalytic systems.

Raman spectroscopy provides complementary vibrational evidence for this transformation (Figure 2i). Prior to annealing, the spectrum is dominated by weak, broadened features associated with residual metallic components and poorly ordered oxide species. After annealing, distinct Raman bands characteristic of β-Ga_2_O_3_ emerge, corresponding to Ga–O stretching and bending modes, accompanied by additional bands attributable to Gd–O vibrations.^41,42^ Raman mode sharpening and intensity redistribution indicate reduced vibrational disorder and increased lattice coherence, in agreement with the strain reduction derived from Williamson–Hall analysis. The absence of Raman signatures associated with bulk, phase-segregated Gd_2_O_3_ further suggests that gadolinium is present predominantly in nanometric or interfacial oxide environments rather than as large, isolated oxide crystallites. Figure 2 demonstrates that thermal annealing converts the Galinstan-Gd precursor into oxide-dominated material that provides a robust foundation for the heterointerface analysis presented in Figure 3.

### 2.2 Atomic-Scale Growth and Heterointerface Characterization

Figure 3 resolves the local growth behavior, structural identity, and crystallographic coherence of gadolinium-derived oxide nanodomains at the nanoparticle surface. At this stage, the analysis shifts from ensemble-averaged characterization to site-specific, atomic-scale observations aimed at elucidating how Gd segregates, oxidizes, and crystallizes within the Ga–In–O matrix. Low- and intermediate-magnification HRTEM images (Figure 3a) reveal high-contrast nanodomains localized at the particle surface following annealing. These domains exhibit faceted morphologies and enhanced lattice contrast relative to the surrounding Ga–In–O matrix, consistent with the formation of a secondary crystalline oxide phase rather than compositional fluctuations within β-Ga_2_O_3_. Their confinement to the near-surface region indicates that gadolinium oxidation and crystallization proceed preferentially at the oxide-environment interface rather than within the particle core. High-magnification HRTEM imaging (Figure 3b) resolves well-defined lattice fringes within these nanodomains, with an interplanar spacing of ∼1.91 Å extracted from line-profile analysis. This spacing closely matches reported values for the {440} planes of cubic c-Gd_2_O_3_ (*a* ≈ 10.8 Å),^43,44^ providing strong crystallographic evidence that the gadolinium-rich domains adopt a cubic oxide structure rather than a distorted or amorphous configuration, and indicating coherent growth over several nanometers once nucleated. To directly correlate lattice contrast with composition, atomic-scale EDS mapping of the same regions (Figure 3c) shows spatially colocalized Gd and O signals overlapping with the high-contrast lattice regions, while Ga is comparatively depleted. This compositional contrast confirms that the crystalline domains correspond to gadolinium-rich oxide nanodomains rather than Ga–O lattice distortions or projection artifacts, and further indicates that Gd does not substitute uniformly into the β-Ga_2_O_3_ lattice at this stage but forms structurally distinct oxide motifs embedded within the Ga–In–O host.

Additional crystallographic insight is obtained from atomic-scale imaging of higher-order lattice planes (Figure 3c, lower panel), where fringes corresponding to the (440) family of planes are resolved. The measured interplanar spacing again converges to ∼1.91 Å, reinforcing the assignment to c-Gd_2_O_3_ and demonstrating internal crystallographic consistency across multiple lattice directions. The accompanying schematic overlay highlights the correspondence between the observed fringe orientations and the expected atomic arrangement of well-defined cubic Gd_2_O_3_ nanodomains. Reciprocal-space confirmation is provided by FFT analysis of the HRTEM images (Figure 3d), which exhibits distinct diffraction spots indexed to the {440} family of reflections of c-Gd_2_O_3_. Given the nanodomain size and the presence of diffuse ring intensity, this FFT is interpreted in terms of reflection-family indexing rather than a strict zone-axis assignment, thereby avoiding overinterpretation of the orientation relationship. The presence of symmetry-related {440} reflections supports three-dimensional crystallinity of the gadolinium oxide nanodomains rather than purely disordered or amorphous order. Notably, these FFT features are absent in regions dominated by β-Ga_2_O_3_, confirming that the gadolinium oxide nanodomains constitute a structurally distinct phase rather than a strained extension of the Ga–O lattice. To support these assignments, the cubic Gd_2_O_3_ structure was modeled using Materials Studio and VESTA (Figure 3e), with simulated lattice planes reproducing the observed fringe spacings and supporting the {440} reflection-family assignment.

Beyond structural imaging, vibrational and diffraction spectroscopies were employed to probe heterointerface-sensitive signatures of the nanodomains. Raman spectra acquired before and after annealing (Figures 3f and S3) show systematic changes in peak position and line width: after annealing, Gd–O-related modes become more pronounced and sharper, with reduced full width at half-maximum (fwhm), indicating decreased vibrational disorder and increased lattice coherence within the Gd_2_O_3_ domains. The observed intensity enhancement (ΔΔ) further suggests an increase in the population or crystallinity of Gd–O environments rather than simple surface adsorption. Normalized Raman intensity comparisons across multiple vibrational modes (Figure 3g) quantitatively confirm this trend, showing consistent intensity increases for Gd–O-related modes after annealing. These spectroscopic changes mirror the crystallographic ordering observed by HRTEM and FFT. Complementary XRD analysis (Figure 3h) provides ensemble-scale confirmation, with reflections corresponding to c-Gd_2_O_3_ planes, including (222), (440), and (611), becoming more defined after annealing and exhibiting peak sharpening indicative of strain relaxation. Importantly, these reflections do not dominate over the β-Ga_2_O_3_ matrix peaks, consistent with the nanoscale, surface-localized nature of the gadolinium oxide phase. The apparent differences between the diffraction features in Figures 2h and 3h arise from the fundamentally different structural sensitivities of ensemble-averaged XRD versus local crystallographic probes. While bulk XRD (Figure 2h) is dominated by the β-Ga_2_O_3_ matrix and therefore weakly reflects the low-volume-fraction Gd_2_O_3_ phase, the postannealing pattern in Figure 3h, interpreted alongside HRTEM, FFT, and Raman data, captures the emergence of locally crystallized c-Gd_2_O_3_ nanodomains. Micro-Raman spectroscopy further confirms this local ordering through phonon sharpening and intensity redistribution, even where long-range coherence remains insufficient to dominate bulk diffraction. Figure 3 demonstrates that while the Gd_2_O_3_ nanodomains resolved here are crystalline, their nanoscale dimensions and sharp interfaces with the surrounding Ga–In–O matrix necessarily give rise to a high density of under-coordinated Gd–O sites at domain boundaries. At these heterointerfaces, Gd ions are expected to experience coordination environments that deviate from bulk c-Gd_2_O_3_ symmetry, including reduced oxygen coordination numbers and locally distorted bonding geometries. Such interfacial motifs can be viewed as few-atom Gd centers, spatially confined and electronically stabilized by the oxide host rather than existing as extended bulk phases. The coexistence of well-ordered crystalline interiors with structurally relaxed interfaces, as evidenced by FFT coherence and strain reduction, suggests that annealing promotes not only crystallization but also atomic-scale site definition at Ga–O/Gd-O junctions. This structural scenario is interesting, where catalytic functionality is often governed by isolated or low-coordination metal centers embedded within an inorganic matrix rather than by bulk oxide phase.

### 2.3 XPS Studies and Characterization

Figure 4 examines the chemical-state evolution of the Galinstan-Gd nanopowders induced by thermal annealing, with particular emphasis on the coupled behavior of Ga, Gd, In, and O at the near-surface region. XPS measurements were performed before and after annealing to track changes in metal–oxygen coordination, defect populations, and valence-band electronic structure as the system transitions from a liquid-metal-derived, nonequilibrium oxide to a structurally and electronically stabilized composite oxide. Prior to annealing, the O 1s spectrum (Figure 4a, top) is composed of multiple contributions centered at approximately 530.4, 531.5, and ∼533.0 eV, corresponding to lattice oxygen, defect-related oxygen states, and surface hydroxyl species, respectively.^45,46^ The significant intensity of the defect-related and hydroxyl components indicates incomplete oxygen coordination and a high density of oxygen-vacancy–associated environments, consistent with rapid oxidation during sonication-driven nanoparticle formation.^47^ After annealing (Figure 4a, bottom), the O 1s envelope redistributes markedly: the lattice oxygen component becomes dominant and shifts slightly to lower binding energy (∼530.2 eV), while the defect-related and hydroxyl contributions are reduced.^46^ This evolution reflects progressive incorporation of oxygen into stoichiometric metal–oxygen lattices and suppression of loosely bound or vacancy-associated oxygen species. The Ga 3d spectra (Figure 4b) show correlated changes. Before annealing, the Ga 3d region is characterized by a Ga–O lattice peak near ∼19.95 eV accompanied by a broader, higher-binding-energy component associated with heterogeneous or strained Ga–O coordination. Following annealing, the Ga 3d peak sharpens and shifts toward lower binding energy (∼18.80 eV), indicating stabilization of Ga^3+^ within a more uniform Ga–O lattice environment characteristic of β-Ga_2_O_3_. No additional Ga oxidation states are detected, suggesting that annealing primarily reorganizes existing Ga–O coordination rather than driving further oxidation. In parallel, the In 3d spectra (Figure 4c) evolve from relatively broad features before annealing to narrower and more symmetric doublets after annealing. The In 3d_5/2_ peak shifts modestly (from ∼444.82 to ∼443.80 eV), reflecting improved homogeneity of the In coordination environment. Importantly, the binding energies remain consistent with metallic or weakly oxidized indium, indicating that In does not fully convert into a bulk oxide phase but instead remains partially metallic or interfacial within the oxide matrix. This behavior aligns with XRD observations of residual metallic components and highlights the composite nature of the Galinstan-derived system. The Gd 3d spectra (Figure 4d) provide direct insight into the chemical state of gadolinium. Before annealing, the Gd 3d features are broad and accompanied by pronounced satellite structures,^48^ indicative of chemically heterogeneous Gd–O environments associated with incipient oxide formation.^49^ After annealing, the Gd 3d_5/2_ and 3d_3/2_ components become sharper and better resolved, with binding energies and satellite structures characteristic of Gd^3+^ in an oxide lattice. No signatures of reduced Gd species or mixed-valence states are observed, indicating that gadolinium undergoes selective oxidation and stabilization rather than redox cycling during annealing.

**Figure 4:**
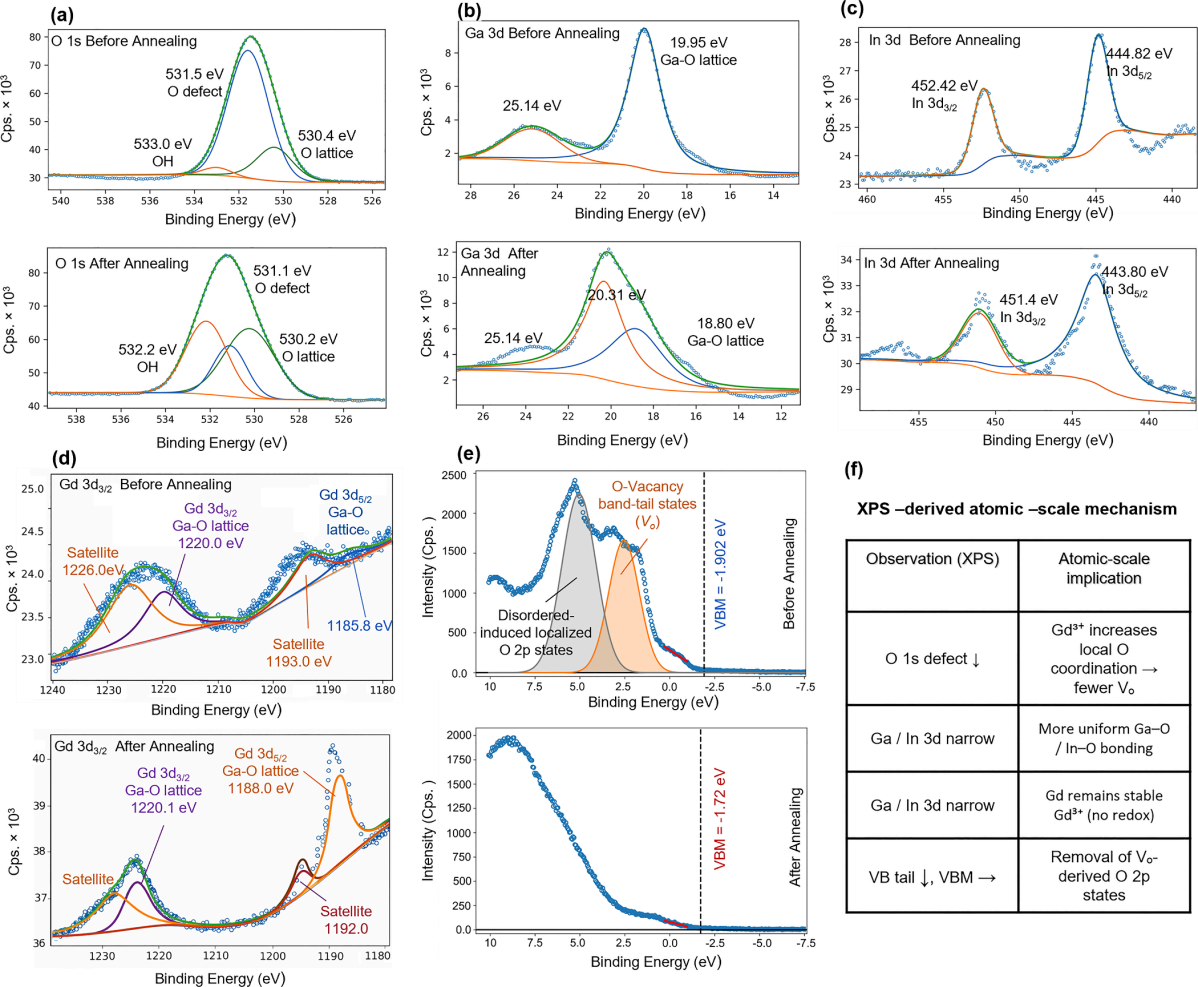
(a) XPS evidence of annealing-induced defect suppression and chemical stabilization. (a) O 1s spectra showing reduced hydroxyl and defect components and enhanced lattice oxygen after annealing. (b,c) Ga 3d and In 3d core levels narrow with minimal binding-energy shifts, indicating more uniform metal–oxygen coordination without redox changes. (d) Gd 3d spectra confirm stable Gd^**3**+^ before and after annealing with reduced satellite intensity. (e) Valence-band spectra reveal suppression of O 2p defect-tail states and a shift of the valence-band maximum toward lower binding energy. (f) Summary linking defect passivation, stabilized Gd^**3**+^ coordination, and preserved bulk electronic structure.

Changes in metal–oxygen coordination are reflected directly in the valence-band spectra (Figure 4e). Before annealing, the valence band exhibits a pronounced tail extending toward the Fermi level, attributed to oxygen-vacancy-derived O 2p states and disorder-induced localized electronic states. The extracted valence-band maximum (VBM) is located at approximately 1.90 eV. After annealing, this defect-related tail is strongly suppressed and the VBM shifts to ∼1.72 eV, indicating removal of vacancy-associated electronic states and refinement of the near-surface electronic structure.^50^ This electronic evolution is consistent with the reduced O 1s defect contribution and the overall chemical ordering induced by annealing. Taken together (Figure 4f), the XPS results provide a chemically resolved picture of how thermal annealing transforms the Ga–Gd Nanoparticles (NPs) from a structurally heterogeneous, defect-rich surface into a chemically and electronically refined oxide system. Rather than evolving independently, Ga, Gd, In, and O undergo a concerted reorganization of metal–oxygen coordination and electronic structure, driven by the simultaneous formation of a β-Ga_2_O_3_ matrix and surface-localized Gd_2_O_3_ nanodomains. Annealing suppresses disorder-driven oxygen states, stabilizes Ga–O coordination, homogenizes In environments without full oxidation, and converts Gd into chemically stable Gd^3+^ oxide motifs. This reorganization is accompanied by removal of oxygen-vacancy–derived valence-band states and refinement of the electronic landscape. These changes yield coordination-defined Ga–O/Gd-O heterointerfaces embedded within an electronically equilibrated oxide framework.

### 2.4 UPS and NMR Probing of Nanostructrues

Figure 5 interrogates the electronic alignment and local atomic environment of the Galinstan–Gd nanostructures using ultraviolet photoelectron spectroscopy (UPS) and solid-state ^69^Ga/^71^Ga NMR. These measurements provide complementary perspectives to XPS: UPS is sensitive to surface dipoles, work function, and band alignment, while NMR probes local coordination and magnetic relaxation of Ga nuclei in the presence of paramagnetic Gd species. The UPS spectra recorded before and after thermal annealing (Figure 5a,b) show a clear and reproducible modification of the surface electronic alignment. Prior to annealing, the spectra indicate a relatively low work function (Φ ≈ 4.52 eV), accompanied by pronounced spectral features associated with defect-induced surface dipoles. The Fermi level appears pinned, consistent with the presence of oxygen-vacancy–derived states near the valence-band edge,^35^ as independently observed in the XPS valence-band measurements. After annealing, the UPS cutoff shifts toward higher binding energy, corresponding to an increased work function (Φ ≈ 4.92 eV). This change is accompanied by a marked reduction in spectral intensity associated with defect-related states, indicating partial unpinning of the Fermi level. Importantly, the relative position between the Fermi level and the VBM remains essentially unchanged, demonstrating that the bulk electronic structure is preserved. The observed shift is therefore attributed to a modification of the vacuum level (Δ*E*_vac_) rather than to band bending or bulk doping effects. The schematic in Figure 5c illustrates this mechanism explicitly: annealing suppresses defect-induced surface dipoles, reducing their contribution to the vacuum-level offset and resulting in an increased work function. This interpretation is fully consistent with the XPS-derived reduction in O 1s defect components and the suppression of valence-band tail states, reinforcing the conclusion that surface defect passivation, not bulk electronic restructuring, governs the UPS response.

**Figure 5:**
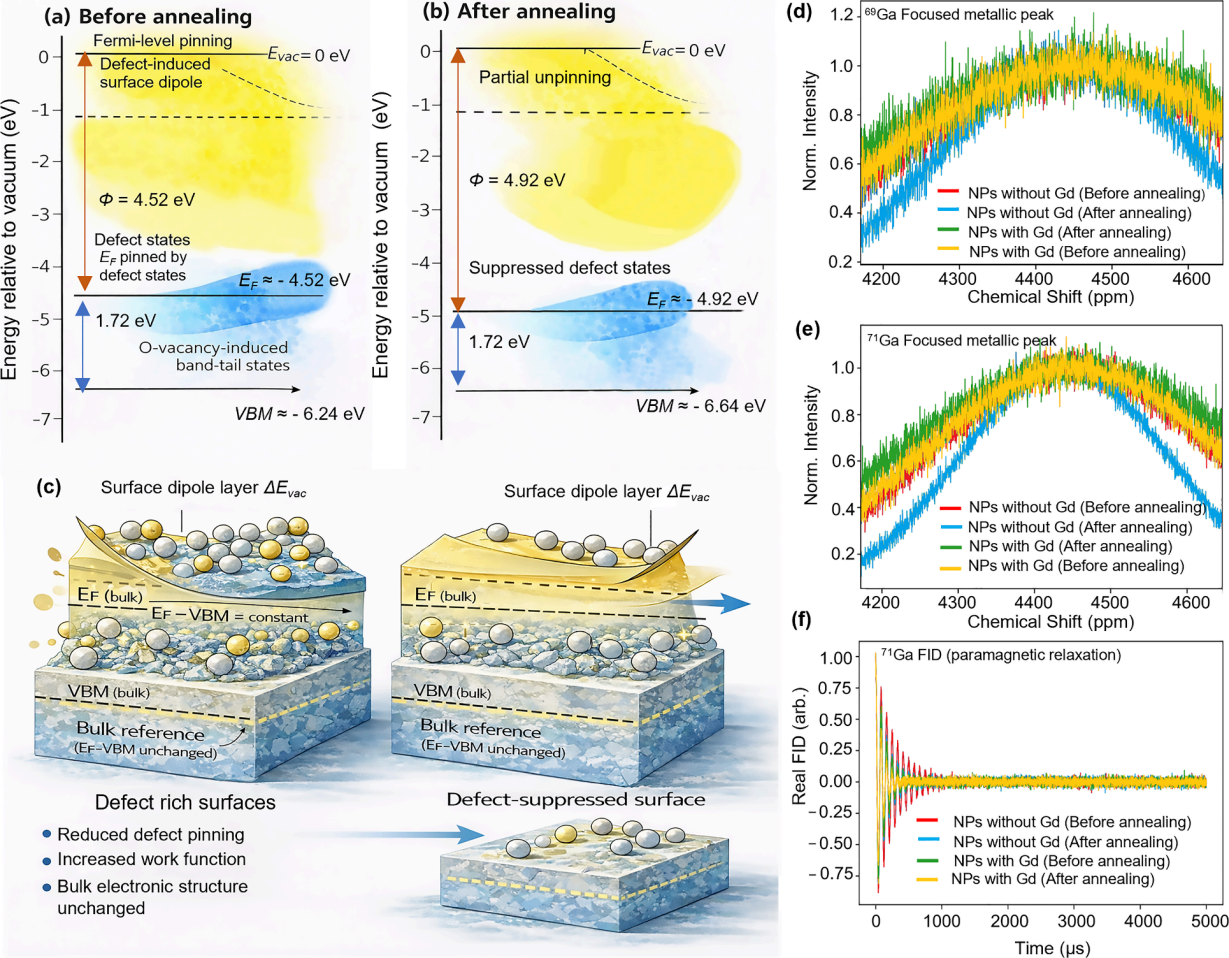
Electronic structure evolution and magnetic signatures of Ga–Gd oxide nanocomposites upon annealing. (a,b) UPS-derived energy-level diagrams before and after annealing, showing defect-induced Fermi-level pinning and low work function prior to annealing (Φ ≈ 4.52 eV), followed by defect suppression, partial Fermi-level unpinning, and an increased work function (Φ ≈ 4.92 eV) governed by a vacuum-level shift (Δ*E*_Vac_), while bulk band alignment remains unchanged. (c) Schematic illustrating relaxation of the surface dipole layer upon defect passivation, resulting in reduced surface pinning and preserved bulk electronic structures. (d,e) ^69^Ga and ^71^Ga solid-state NMR spectra centered on metallic Ga resonances, showing line width narrowing and reduced inhomogeneous broadening after annealing, consistent with improved local ordering. (f) ^71^Ga free-induction decay (FID) traces revealing accelerated paramagnetic relaxation in Gd-containing samples, enhanced after annealing, consistent with localized magnetic interactions rather than chemical substitution of Ga sites.

The ^69^Ga and ^71^Ga MAS NMR spectra^51^ (Figure 5d,e), together with the liquid-state NMR data in Figures S4–S7, provide detailed insight into the local Ga environments and their magnetic response to annealing and Gd incorporation. High-field liquid-state ^69^Ga NMR (Figure S4) reveals a well-defined metallic Ga resonance centered near ∼4460 ppm, with a secondary, lower-shift contribution (∼220 ppm) attributed to surface-like or partially oxidized Ga species. Thermal annealing leads to modest line width narrowing in Gd-free samples, consistent with reduced structural disorder, whereas Gd-containing samples exhibit systematic line width broadening and reduced coherence. These effects are further amplified under low-field conditions (Figure S5), where enhanced motional and quadrupolar broadening accentuate the magnetic relaxation contribution from Gd^3+^ centers.

Isotope-resolved comparison using ^71^Ga NMR (Figures S6 and S7) clarifies the origin of these effects. Relative to ^69^Ga, ^71^Ga exhibits intrinsically narrower resonances due to reduced second-order quadrupolar broadening, yet still shows pronounced relaxation acceleration in the presence of Gd, particularly after annealing. The field-dependent behavior, stronger coherence at high field and increased broadening at low field, confirms that the dominant interaction is paramagnetic relaxation rather than chemical shift dispersion. Crucially, across all fields and isotopes, the peak positions remain essentially invariant, demonstrating that Ga does not undergo a change in oxidation state or coordination symmetry. Instead, the dominant effect of Gd is to introduce localized magnetic fluctuations that shorten T_2_* and broaden Ga resonances through dipolar and contact interactions. Further evidence for this interpretation is provided by the ^71^Ga free-induction decay (FID) measurements (Figure 5f), where Gd-containing samples show markedly faster signal decay than their Gd-free counterparts. This paramagnetic relaxation effect intensifies after annealing, consistent with the formation of structurally coherent Gd_2_O_3_ nanodomains that bring Ga environments into closer magnetic proximity with Gd^3+^ centers. The absence of new resonances or large chemical-shift changes confirms that Gd influences Ga primarily through magnetic and electronic interactions rather than direct chemical substitution into the Ga lattice.^35,52^

Integrated XPS, UPS, and NMR analyses demonstrate that thermal annealing induces a concerted reorganization of chemical coordination, surface electrostatics, and local magnetic environments in the Ga–Gd NPs. XPS reveals that annealing suppresses oxygen-vacancy–related and hydroxyl-derived O 1s components while narrowing Ga 3d and In 3d core levels, indicating homogenization of Ga–O and In–O coordination without redox-driven oxidation-state changes. In parallel, sharpening of the Gd 3d features confirms stabilization of Gd^3+^ within well-defined oxide environments, consistent with the formation of surface-localized Gd_2_O_3_ nanodomains rather than uniform lattice substitution. These chemical refinements translate directly into surface electronic restructuring observed by UPS, where defect suppression relaxes the surface dipole layer, increases the work function, and partially unpins Fermi level without altering the bulk Fermi level-valence band alignment, indicating a vacuum-level-dominated effect confined to near-surface region. Solid-state ^69^Ga/^71^Ga NMR further shows that metallic Ga environments persist after annealing, while Gd-containing samples exhibit pronounced line width broadening and accelerated relaxation, reflecting localized magnetic interactions between Ga nuclei and nearby paramagnetic Gd^3+^ centers rather than chemical substitution.

### 2.5 Magnetically Modulated Electrochemical CO_2_ Conversion

Figure 6 examines how thermally induced structural ordering activates magnetic-field sensitivity during electrochemical CO_2_ conversion on Ga–Gd NP electrodes. The figure integrates the magneto-electrochemical measurement architecture (Figure 6a,b), zero-field and field-applied cyclic voltammetry (CV) responses (Figure 6c–e), and potential-resolved magnetic-response metrics (Figure 6f–h). The experimental configuration (Figure 6a,b) combines electrochemical control (CV/CA/EIS), external magnetic-field modulation (*B* = 0–200 mT), and downstream gas-phase detection within a sealed flow cell. The Ga–Gd NP layer serves as the working electrode and is positioned directly above an electromagnetic base, ensuring spatially uniform magnetic exposure localized at the electrode–electrolyte interface. This geometry minimizes bulk magnetohydrodynamic contributions and excludes Lorentz-force-driven convection, thereby isolating magnetic effects at the interfacial electrochemical level. CV measurements under CO_2_ at zero magnetic field (*B* = 0) establish the baseline electrochemical behavior. For Galinstan and Ga–Gd electrodes prior to annealing (Figure 6c,d; *B* = 0), the CVs exhibit weak and broadened cathodic features within the CO_2_ interaction window (−0.85 to −0.35 V vs Ag/AgCl), with limited scan-rate dependence. These responses indicate kinetically sluggish CO_2_ activation on a structurally disordered surface. After annealing, the zero-field CV of the Ga–Gd electrode (Figure 6e; *B* = 0) displays a markedly higher cathodic current while preserving the overall CV topology and onset potential. This enhancement is consistent with XPS- and UPS-derived evidence for defect suppression and improved electronic equilibration at the Ga–Gd oxide interface.

**Figure 6:**
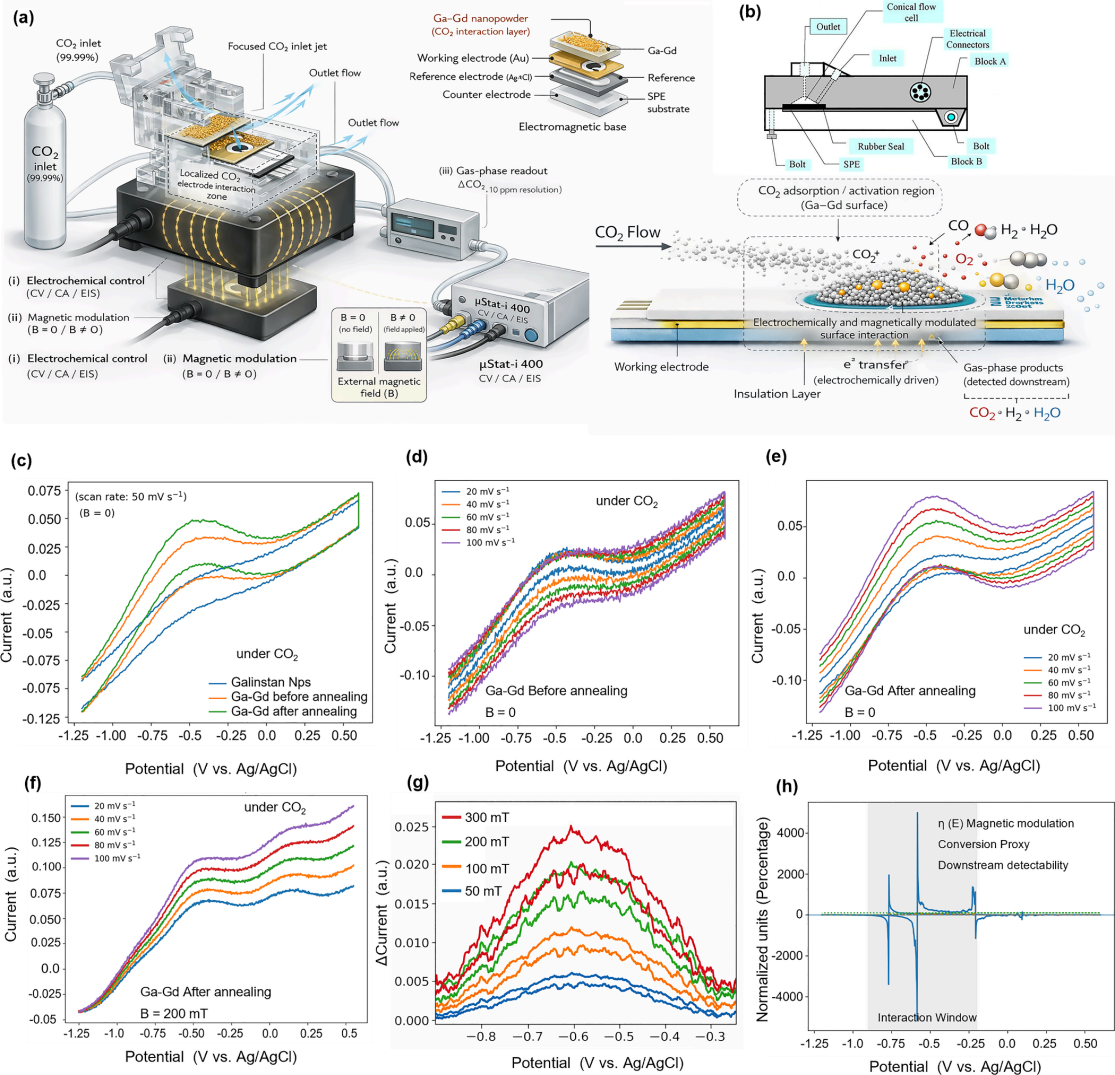
Magneto-electrochemical CO_2_ conversion on annealed Ga–Gd oxide nanocomposites. (a,b) Integrated magneto-electrochemical platform combining controlled CO_2_ flow, a Ga–Gd nanopowder working electrode, electrochemical regulation (CV/CA/EIS), and an external magnetic field applied normal to the electrode surface. (c–e) Zero-field (*B* = 0) CO_2_-saturated cyclic voltammograms establishing baseline electrochemical behavior for Galinstan, Ga–Gd before annealing, and Ga–Gd after annealing, respectively. (f) Cyclic voltammograms of annealed Ga–Gd acquired under an applied magnetic field (*B* = 200 mT). (g) Differential current response (Δ*I*) as a function of magnetic field strength (50–300 mT). (h) Potential-resolved magnetic modulation factor η­(*E*), highlighting the narrow interaction window where magnetic sensitivity maximizes CO_2_ conversion.

Magnetic-field effects emerge meaningfully after annealing and under applied field conditions. When a magnetic field is applied to the annealed Ga–Gd NP electrode (Figure 6f; *B* = 200 mT, Figure S8), a pronounced enhancement of the cathodic current is observed selectively within the CO_2_ interaction window, while the CV shape and reaction onset remain unchanged. Scan-rate-dependent measurements (20–100 mV s^–1^) show that both zero-field and field-on currents scale proportionally with scan rate, indicating faradaic control rather than capacitive artifacts or mass-transport limitations. The magnetic contribution is quantitatively isolated by evaluating the differential current, Δ*I*(*E*) = *I*_(*B* ≠ 0)_ – *I*_(*B*=0)_ (Figures 6g,h, S9, and S10). The resulting Δ*I*(*E*) profile exhibits a pronounced maximum centered at −0.55 ± 0.05 V vs Ag/AgCl, coinciding with the region of steepest CO_2_ reduction current. Outside this window, Δ*I* collapses to zero, demonstrating that magnetic sensitivity is strictly reaction-specific and does not affect background electrochemistry. Field-dependent measurements reveal a monotonic increase in peak Δ*I* with increasing *B*, reaching ∼0.008–0.010 au at *B* ≥ 0.5 T before approaching saturation. The half-maximum response occurs at relatively low fields (∼0.15–0.25 T), well below the regime where bulk magnetic forces would be expected to dominate. The absence of hysteresis upon magnetic-field cycling and the strict confinement of Δ*I* to the CO_2_ activation regime are inconsistent with magnetohydrodynamic or mechanical artifacts.^53^ These observations demonstrate that magnetic modulation of CO_2_ electroreduction is an emergent interfacial property activated only after annealing produces a chemically ordered, defect-suppressed Ga–Gd oxide interface. The magnetic field selectively enhances interfacial charge-transfer kinetics without altering reaction onset or pathway, pointing to a localized, electronically mediated mechanism. These findings motivate examination of the field-sensitive intermediate and its spin-dependent stabilization in the following section.

### 2.6 Quantitative Analysis and Spectroscopic Validation of CO_2_ Conversion

Figure 7 provides the quantitative and spectroscopic validation of magnetically modulated CO_2_ electroreduction on Ga–Gd electrodes by integrating electrochemical performance metrics with gas-phase spectroscopy. Whereas Figures 4–6 established that thermal annealing produces a defect-suppressed and magnetically responsive Ga–Gd interface with field-sensitive electrochemical kinetics, Figure 7 directly links this interfacial state to selective CO and CH_3_OH formation while excluding competing reduction pathways. To strengthen the quantitative interpretation and explicitly address hydrogen utilization, product quantification, selectivity, and operational stability, additional Supporting Information Figures S11–S18 are incorporated, and stability metrics are included in the revised analysis. Figure 7a summarizes the overall CO_2_ conversion efficiency measured under zero magnetic field and under an applied magnetic field (*B* = 200 mT) for Galinstan and Ga–Gd electrodes before and after annealing. All CO_2_ conversion efficiencies, product formation rates, and Faradaic efficiencies reported in Figures 7 and 8 were determined under steady-state chronoamperometric (CA) operation at fixed potential after current stabilization; cyclic voltammetry (CV) was used only to assess onset potentials and magnetic-field-induced changes in electrochemical kinetics (Figures 6 and S8–S10), not for product quantification. Table 1 summarizes the key quantitative electrochemical metrics for CO_2_ electroreduction under controlled-potential chronoamperometry, including CO_2_ conversion efficiency, CO and CH_3_OH formation rates, Faradaic efficiency, and magnetic-field enhancement. These values provide a consolidated comparison of magnetic modulation effects extracted from the data presented in Figures 7 and 8.

**Figure 7:**
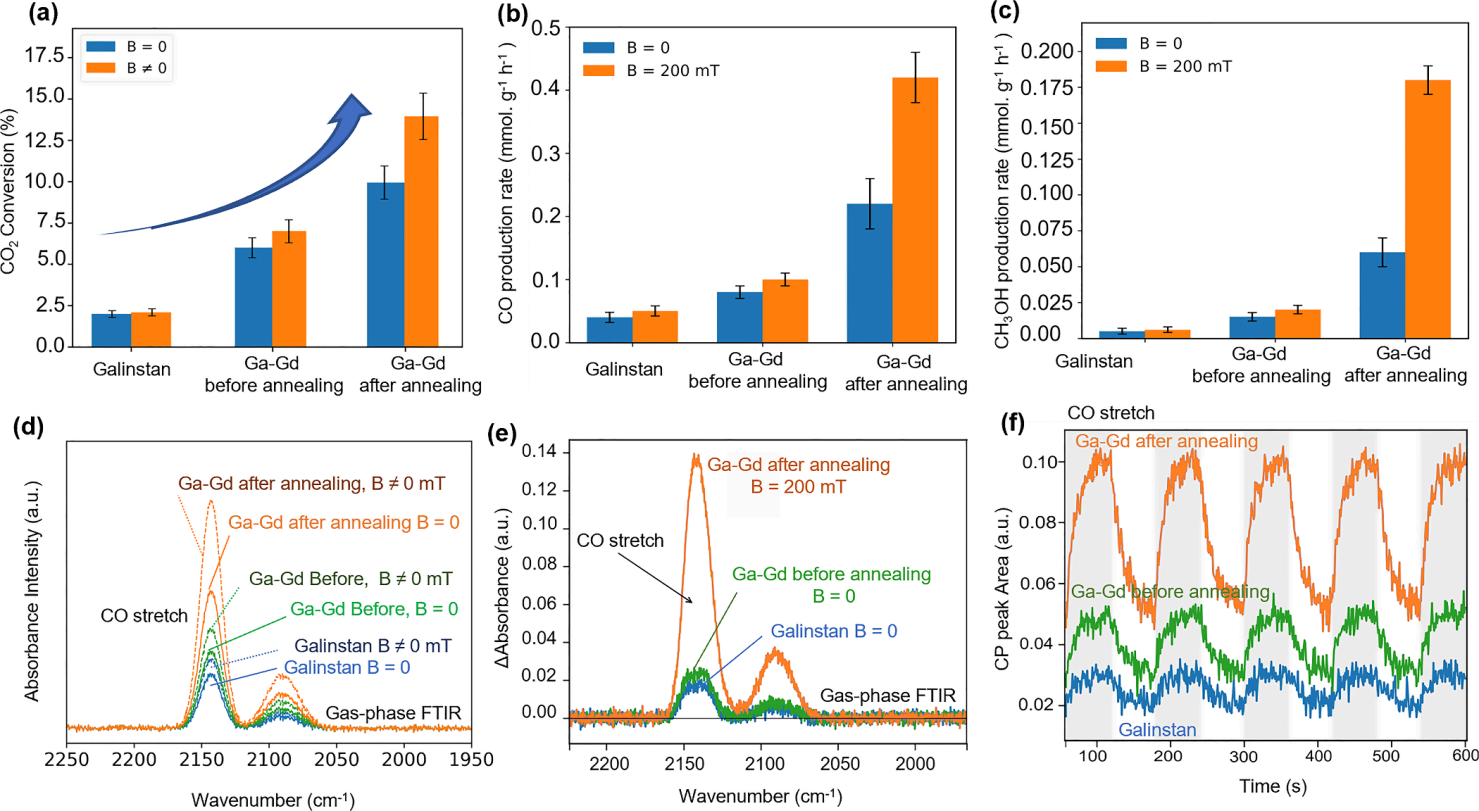
Quantitative CO_2_RR performance and gas-phase identification of CO under magnetic field. (a–c) CO_2_ conversion, CO formation rate, and CH_3_OH formation rate measured by steady-state chronoamperometry (CA) at *B* = 0 and *B* = 200 mT for Galinstan and Ga–Gd electrodes before/after annealing. (d) Gas-phase FTIR CO-stretch region for six catalyst/field states. (e) Representative CO spectra highlighting selective enhancement for annealed Ga–Gd. (f) Time-resolved integrated CO peak area during electrolysis cycling.

**Figure 8:**
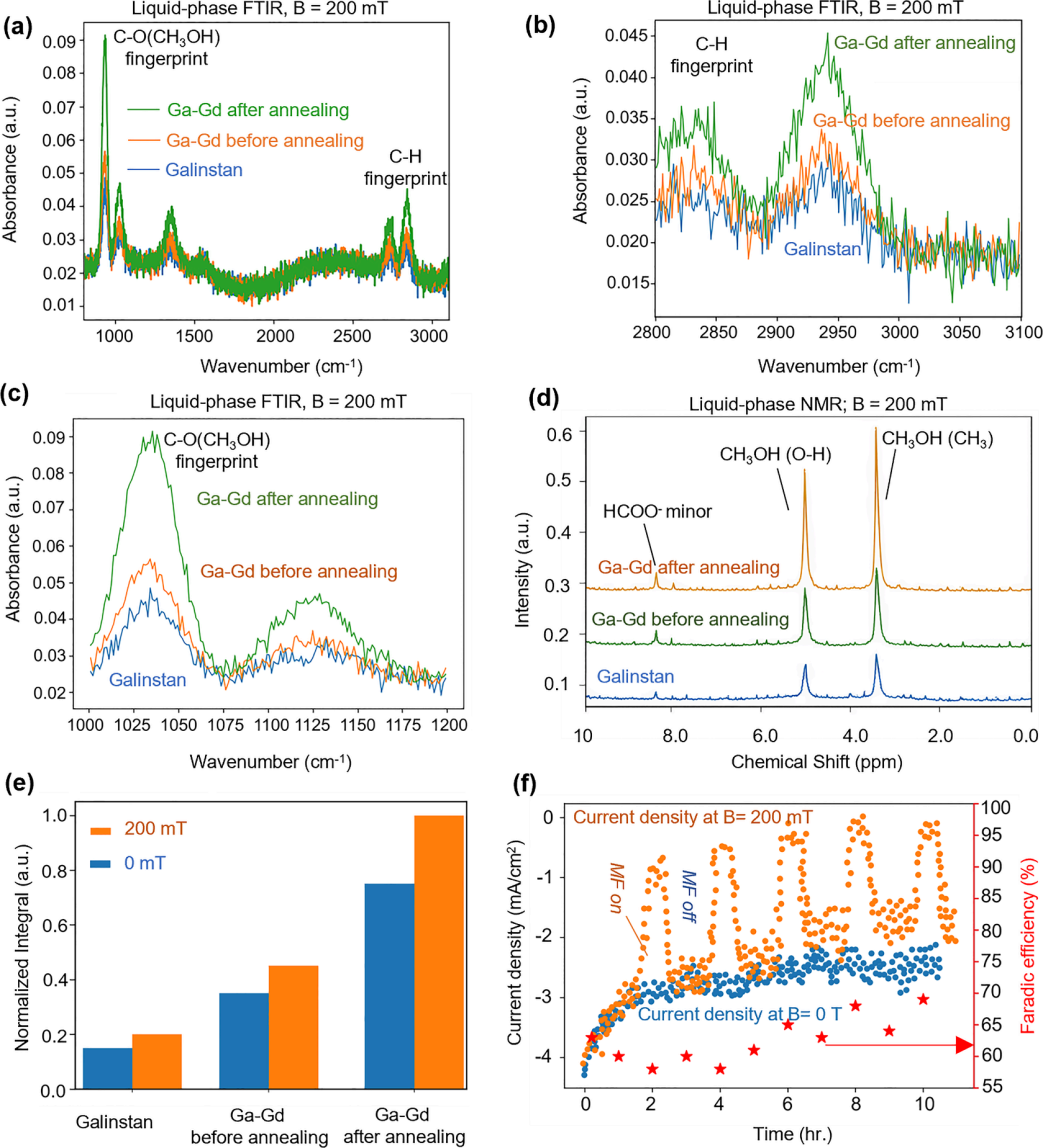
Liquid-phase validation of CH_3_OH formation and CA stability under magnetic field. (a–c) Liquid-phase FTIR spectra highlighting methanol fingerprints (C–O and C–H) after CO_2_RR at *B* = 200 mT. (d) Liquid-phase ^1^H NMR confirming CH_3_OH as the dominant liquid product (minor HCOO^–^). (e) Normalized methanol-related integrals at *B* = 0 and *B* = 200 mT. (f) 10 h CA showing stable current density and stable combined Faradaic efficiency (CO + CH_3_OH) with reversible field-induced modulation.

**Table 1:** Quantitative Electrochemical Metrics of Magnetic Modulation during CO_2_ Reduction

parameter	Ga–Gd (before annealing)	Ga–Gd (after annealing, B = 0)	Ga–Gd (after annealing, B ≠ 0)	**key**observation
CO_2_ interaction window (V vs Ag/AgCl)	–0.85 to −0.35	–0.85 to −0.35	0.85 to −0.35	identical potential window across all states
peak cathodic current, *I*_p_ (a.u.)	∼0.030–0.035	∼0.045–0.050	∼0.060–0.070	annealing increases baseline activity; magnetic field selectively amplifies
differential magnetic current, Δ*I*_max_ (a.u.)	≤0.001		∼0.008–0.010	strong Δ*I* emerges only after annealing
relative enhancement factor, *I*(*B* ≠0)/*I*(*B* = 0)	∼1.02		∼1.35–1.50	nonlinear, field-enabled enhancement
potential of maximum Δ*I* (V vs Ag/AgCl)			–0.55 ± 0.05	coincides with maximum CO_2_ reduction slope
scan-rate dependence (20–100 mV s^–1^)	weak, featureless	proportional scaling	proportional scaling + Δ*I* preserved	magnetic effect is kinetic, not capacitive
field for half-max response, *B*_1/2_ (T)			∼0.15–0.25 T	subtesla sensitivity
Δ*I* saturation regime			≥0.5 T	suggests finite interfacial magnetic coupling
hysteresis (B on/off)	none	none	none	rules out magnetohydrodynamic artifacts
Δ*I* outside CO_2_ window	∼0	∼0	∼0	magnetic effect is reaction-specific

Bare Galinstan exhibits negligible conversion (<2%) and shows no statistically significant magnetic response. Incorporation of Gd prior to annealing leads to a modest increase in conversion (∼5–6%) while retaining weak field dependence. In contrast, annealed Ga–Gd displays a pronounced magnetic enhancement, with CO_2_ conversion increasing from approximately 10% at *B* = 0 to ∼14–15% under applied field, corresponding to an enhancement factor of ∼1.4–1.5. This behavior is fully consistent with the magnetic differential current (Δ*I*) quantified in Figure 6 and confirms that magnetic sensitivity emerges only after annealing-induced interfacial ordering.

Product-resolved formation rates clarify the origin of this enhancement. As shown in Figure 7b, the CO production rate increases systematically with annealing and magnetic field, reaching ∼0.42 mmol g^–1^ h^–1^ for annealed Ga–Gd under *B* = 200 mT compared with ∼0.22 mmol g^–1^ h^–1^ at zero field. An even stronger magnetic response is observed for CH_3_OH formation (Figure 7c), where the production rate increases from ∼0.06 mmol g^–1^ h^–1^ at *B* = 0 to ∼0.18 mmol g^–1^ h^–1^ under applied field. Normalized CO vs CH_3_OH selectivity derived from the CA-based formation rates is provided in Supporting Information Figure S11. The stronger magnetic sensitivity of CH_3_OH relative to CO indicates that the applied field preferentially influences later-stage, multielectron and hydrogen-coupled reduction steps rather than the initial CO_2_ activation process, consistent with prior operando FTIR and electrochemical studies.^54–56^ Neither Galinstan nor Ga–Gd before annealing exhibits comparable magnetic amplification, confirming that magnetic-field sensitivity arises only once the Ga–Gd interface is electronically stabilized, in agreement with UPS-derived surface dipole relaxation and NMR-resolved magnetic coupling. This interpretation is reinforced by converging electrochemical and spectroscopic observations. Magnetic enhancement is strictly confined to the CO_2_ reduction potential window, with no shift in onset potential and no emergence of new features in cyclic voltammograms (Figure 6 and Supporting Information Figures S8–S10), indicating that the applied field does not introduce additional electrochemical pathways.

Gas-phase FTIR spectra acquired downstream under *B* = 200 mT (Figure 7d–f) provide direct evidence for CO as the dominant volatile product. Figure 7d compares the absolute CO stretching intensities for six catalyst states (Galinstan, Ga–Gd before annealing, and Ga–Gd after annealing, each at *B* = 0 and *B* = 200 mT), revealing a pronounced magnetic-field-induced enhancement only for annealed Ga–Gd. To isolate the intrinsic magnetic effect, Figure 7e focuses on three representative conditions, annealed Ga–Gd at *B* = 200 mT, Ga–Gd before annealing at *B* = 0, and Galinstan NPs at *B* = 0, highlighting the selective amplification of the CO stretching mode at 2140–2160 cm^–1^. No detectable signatures of CH_4_, C_2_H_4_, or other hydrocarbons are observed, and the absence of gas-phase formate or carbonate features rules out parasitic decomposition pathways. Time-resolved FTIR measurements (Figure 7f) track the integrated CO peak area during repeated on/off electrolysis cycles and demonstrate stable, periodic CO evolution synchronized with current response. The reproducibility of the magnetic enhancement over multiple cycles confirms that the field-induced increase in CO formation is reversible and not associated with catalyst restructuring or transient adsorption effects. Quantitative integration of the CO FTIR peak area (Supporting Information Figure S12) independently confirms the magnetic-field-induced enhancement of CO formation and closely mirrors the CA-derived production-rate trends in Figure 7b.

Liquid-phase FTIR and ^1^H NMR analyses further reveal selective amplification of methanol-related signatures under magnetic field while preserving product identity, with no evidence for new gas, or liquid-phase products. Moreover, the magnetic-field-dependent current response (Δ*I*) is monotonic, reversible, and stable with respect to field cycling and time, excluding mass-transport, hydrodynamic, or thermal artifacts. Liquid-phase FTIR spectra collected after electrolysis (Figure 8a–c) reveal clear vibrational fingerprints of methanol, including C–H stretching modes (2800–3100 cm^–1^) and C–O stretching features (1000–1200 cm^–1^). These features are strongest for annealed Ga–Gd, weaker for preannealed Ga–Gd, and minimal for Galinstan. Expanded spectra and material-resolved comparisons are provided in Figures S11 and S13–S15. Baseline-corrected integration of the C–H and C–O bands (Figures S16 and S17) demonstrates proportional enhancement of hydrogenated liquid products under magnetic field, providing quantitative confirmation beyond peak-intensity comparisons. Complementary liquid-phase ^1^H NMR measurements (Figure 8d,e) provide unambiguous molecular identification and quantification of methanol. The characteristic CH_3_OH resonances at ∼3.3 ppm and ∼4.8 ppm increases systematically with annealing and applied magnetic field, while only trace formate signals are detected. Integrated NMR peak areas (Supporting Information Figure S18) closely follow the CH_3_OH production rates in Figure 7c and the liquid-phase FTIR integrations, confirming that magnetic enhancement reflects genuine methanol formation rather than spectroscopic artifacts or transient adsorption. Figure 8f further extends this analysis by demonstrating long-term catalytic and magnetic stability under continuous operation. Chronoamperometry recorded over 10 h shows stable current density, while simultaneously measured Faradaic efficiency remains constant within experimental uncertainty. Importantly, the magnetic-field-induced current enhancement persists throughout the measurement, and Δ*I* remains constant with time, confirming the absence of magnetic fatigue, catalyst deactivation, or hysteresis. These results demonstrate that the observed magnetic modulation is robust under prolonged electrolysis. To directly assess whether the Ga–In–Gd system undergoes structural reconstruction during CO_2_ electroreduction, high-resolution XPS spectra of Ga 3d, Gd 3d, In 3d, and O 1s were acquired before and after prolonged catalytic operation (Supporting Information, Figure S19). The binding energies and line shapes of Ga 3d and In 3d remain unchanged after electrolysis, with no emergence of low-binding-energy components associated with metallic Ga^0^ or In^0^, indicating preservation of the oxidized Ga–O and In–O coordination environments. Likewise, the Gd 3d spectra retain characteristic Gd^3+^ peak positions and satellite features, confirming chemical and electronic stability of the Gd-derived magnetic nanodomains under operating conditions. The O 1s spectra exhibit a modest reduction in defect-related contributions following catalysis, consistent with interfacial equilibration rather than degradation or phase transformation, and no carbonate- or bicarbonate-related species are detected. Together with stable current density, invariant Faradaic efficiency, and preserved product selectivity during long-term chronoamperometric operation, these results demonstrate that the Ga–Gd heterointerface remains structurally persistent throughout CO_2_ electroreduction.

The role of hydrogen in the present CO_2_ electroreduction system is therefore clarified by the combined gas-phase FTIR, liquid-phase FTIR, and ^1^H NMR analyses. Because molecular H_2_ is infrared inactive, hydrogen participation is evaluated through its incorporation into reaction intermediates and liquid products rather than direct detection of gaseous H_2_. The absence of additional gas-phase features under magnetic field, together with the enhanced formation of hydrogenated liquid products (Figures 8a–e and S11–S18), demonstrates that hydrogen is preferentially utilized in CO_2_ reduction rather than evolving as molecular hydrogen. Overall, the selectivity and magnetic sensitivity observed in Figures 7 and 8 are consistent with the interfacial picture developed in earlier sections. Within this framework, the magnetic enhancement of CO and CH_3_OH formation arises from field-sensitive interfacial charge- and spin-dependent kinetics at a chemically ordered oxide heterointerface, rather than from reconstruction-driven or transient surface effects, consistent with prior reports on electronically stabilized metal–oxide interfaces.^57–60^

## 3 Conclusions

This study demonstrates that precision-engineered Ga–Gd oxide nanocomposites, formed via controlled thermal annealing, enable magnetic field-assisted CO_2_ electroreduction through ultrafine, surface-localized oxide nanodomains. Structural and spectroscopic analyses show that annealing produces a multiphase β-Ga_2_O_3_/Gd_2_O_3_ interface with suppressed defect states, relaxed surface dipoles, and localized paramagnetic environments, while preserving bulk electronic structure. Under applied magnetic fields, these interfaces exhibit pronounced, potential-selective enhancement of CO_2_ reduction activity without altering reaction onset potentials or product identity. Quantitative FTIR and NMR confirm selective formation of CO and CH_3_OH and exclude competing hydrocarbon pathways, indicating that magnetic effects primarily modulate interfacial charge-transfer kinetics and intermediate populations. Together, these results establish ultrafine oxide nanodomains as precision-defined catalytic motifs whose reactivity can be dynamically regulated by external magnetic fields. More broadly, this work points toward a general strategy for coupling nanodomain-level structural precision approaching single-atom-like control at oxide interfaces, with physical-field modulation to achieve controllable electrochemical catalysis for low-carbon energy conversion.

## 4 Experimental Section and Methods

Galinstan alloy was used as the base material for catalyst preparation. Gadolinium nanoparticles (∼5 nm) were incorporated into Galinstan via ultrasonic-assisted mixing to form Ga–Gd composite nanopowders. Galinstan nanoparticles were first generated by ultrasonication in anhydrous ethanol, followed by the addition of Gd nanoparticles and further sonication to promote surface interaction and galvanic exchange between Galinstan and Gd species. The resulting powders were collected and thermally annealed under an inert atmosphere to induce controlled oxidation and interfacial ordering. Annealing stabilized a β-Ga_2_O_3_ matrix while promoting the formation of surface-localized Gd_2_O_3_ nanodomains, with partial retention of metallic Ga–In domains. Samples before and after annealing are denoted Ga–Gd (before annealing) and Ga–Gd (after annealing), respectively. Structural characterization was performed using transmission electron microscopy. High-resolution TEM, STEM, HAADF imaging, SAED, and atomic-scale EDS mapping were conducted on a JEM-ARM200F NEOARM microscope operated at 200 kV. Lattice spacings were extracted from HRTEM images and corresponding FFTs using DigitalMicrograph, and crystallographic models were generated using VESTA and Materials Studio. X-ray diffraction was carried out on a Rigaku SmartLab diffractometer using Cu Kα radiation (λ = 1.5406 Å) over 10–80° (2θ). Phase identification and strain analysis were performed using reference patterns for β-Ga_2_O_3_, c-Gd_2_O_3_, Gd_3_Ga_5_O_12_, and residual metallic phases, with crystallite size and microstrain estimated via Williamson–Hall analysis. Raman spectroscopy was performed using a Horiba LabRAM ARAMIS system with 532 nm excitation to probe lattice ordering and defect evolution before and after annealing. Surface chemical and electronic states were analyzed by X-ray photoelectron spectroscopy using a Thermo Scientific Theta Probe with a monochromated Al Kα source. High-resolution spectra were collected for Ga 3d, Gd 3d, In 3d, and O 1s regions, with binding energies referenced to C 1s at 284.8 eV. Valence-band spectra were used to determine the valence-band maximum. Ultraviolet photoelectron spectroscopy was performed using He I radiation (21.22 eV) to extract work-function changes from secondary-electron cutoff positions. Solid-state ^69^Ga and ^71^Ga MAS NMR and liquid-state NMR measurements were carried out on a Bruker Avance III spectrometer operating at 400 MHz to probe local Ga environments and magnetic relaxation effects. Field- and isotope-dependent measurements were used to distinguish quadrupolar and paramagnetic relaxation contributions. Liquid-phase ^1^H NMR was used to identify and quantify reaction products using D_2_O as solvent and DSS as an internal reference, with concentrations determined by peak integration against calibration standards.

Electrochemical CO_2_ conversion experiments were performed using a μStat-i 400 (Bi)­potentiostat/galvanostat/impedance analyzer (Metrohm DropSens). Screen-printed gold electrodes (DRP-220BT-U75) incorporating an integrated Ag/AgCl reference electrode and a carbon counter electrode were employed. Measurements were conducted in a magnetic flow cell (DRP-CFLWCL-MAGN), which enabled controlled application of an external magnetic field during electrolysis. The electrolyte consisted of 0.5 M KHCO_3_ aqueous solution saturated with CO_2_ (99.999%) for at least 30 min prior to measurements (pH ≈ 7.2), with continuous CO_2_ flow maintained throughout electrochemical operation. All CO_2_ conversion efficiencies, product formation rates, and Faradaic efficiencies were determined under steady-state chronoamperometry (CA) at fixed applied potential after current stabilization. Cyclic voltammetry (CV) was used only to evaluate onset potentials and magnetic-field-dependent changes in electrochemical kinetics, and was not employed for product quantification. Cyclic voltammetry was performed in the potential range of −1.25 to +0.55 V versus Ag/AgCl at scan rates between 20 and 100 mV s^–1^. Magnetic modulation was evaluated by comparing electrochemical responses acquired at zero field (*B* = 0) and under applied magnetic field (*B* ≠ 0). Differential magnetic currents were calculated as Δ*I*(*E*) = *I*(*B* ≠ 0) – *I*(*B* = 0). A static magnetic field oriented perpendicular to the electrode surface was applied using the integrated magnetic platform of the flow cell, with field strengths varied between 50 and 800 mT. Gas-phase products were analyzed operando using a Thermo Scientific Nicolet iS5 FTIR spectrometer equipped with a DTGS detector and connected downstream of the electrochemical cell. Spectra were collected at a resolution of 4 cm^–1^ with at least 10 scans per spectrum. Carbon monoxide was identified by its characteristic C≡O stretching band in the 2140–2170 cm^–1^ region and quantified by integrating this band area. Liquid-phase products were analyzed using the same FTIR system operated in ATR mode with a ZnSe crystal under identical spectral conditions to identify methanol vibrational features. Methanol formation was quantified primarily by liquid-phase ^1^H NMR spectroscopy, with liquid-phase FTIR serving as complementary confirmation. Integrated spectral areas from FTIR and NMR measurements were used as quantitative proxies for product formation rates, and their consistency with electrochemical trends was verified across multiple characterization techniques. CO_2_ conversion efficiency was evaluated under steady-state electrolysis using inline CO_2_ sensor measurements coupled with controlled gas flow rates and normalized to the Galinstan baseline under otherwise identical electrochemical conditions. Product selectivity was evaluated using normalized carbon-product selectivity rather than absolute Faradaic efficiency. Selectivity was defined as the fraction of detected carbon-containing products (CO and CH_3_OH) such that the combined selectivity sums to 100%. All data were processed using instrument software and Python-based routines for baseline correction, normalization, peak fitting, and statistical analysis. Each measurement was repeated at least three times to ensure reproducibility. Detailed spectral processing procedures, integration methods, and supporting data sets are provided in the Supporting Information.

## Supplementary Material



## Data Availability

The data that supports the findings of this study are available from the corresponding authors upon reasonable request.

## Notes

Additional experimental details, supplementary electrochemical measurements, and extended spectroscopic and structural characterization, including cyclic voltammetry and chronoamperometry data, magnetic-field-dependent electrochemical responses, expanded gas-phase and liquid-phase FTIR spectra, quantitative product analysis and selectivity calculations, ^1H NMR spectra of liquid products, long-term stability measurements, and high-resolution XPS spectra (Ga 3d, Gd 3d, In 3d, and O 1s) acquired before and after CO_2_ electroreduction (PDF)
