# Supplementary material for: Magnetic-Field-Assisted CO2 Electroreduction at Precision-Engineered Ga–Gd Oxide Nanodomain Interfaces
Source: Precis Chem. 2026 Feb 11;4(6):850–66. doi: 10.1021/prechem.5c00435 (PMC13292099; doi:10.1021/prechem.5c00435)
Supplement: Supplementary file 1 [file pc5c00435_si_001.pdf]

Supporting Information

Magnetic-Field–Assisted CO_2 Electroreduction at Precision-Engineered Ga–Gd Oxide Nanodomain Interfaces

*Mohammad Karbalaei Akbari [a, b] *[1], Kumar Shrestha [a,b], Noor Aljammal [c], Alireza Pourvahabi Anbari [b, d], Yanbin Cui [e], Serge Zhuiykov [a, b] **

[a] Department of Solid-State Sciences, Faculty of Science, Ghent University, Krijgslaan 281/S1, B-9000, Ghent, Belgium.
[b] Center for Green Chemistry & Environmental Biotechnology, Ghent University Global Campus, Incheon 21985, South Korea.
[c] Laboratory for Chemical Technology (LCT), Department of Materials, Textiles, and Chemical Engineering, Ghent University, Technologiepark 125, 9052 Ghent, Belgium.
[d] Department of Chemistry, Faculty of Science, Ghent University, Ghent, B-9000, Belgium.
[e] Institute of Process Engineering, Chinese Academy of Sciences, Beijing 100190, China.

[1] Corresponding authors: Mohammad.Akbari@ugent.be; Serge.Zhuiykov@ghent.ac.kr

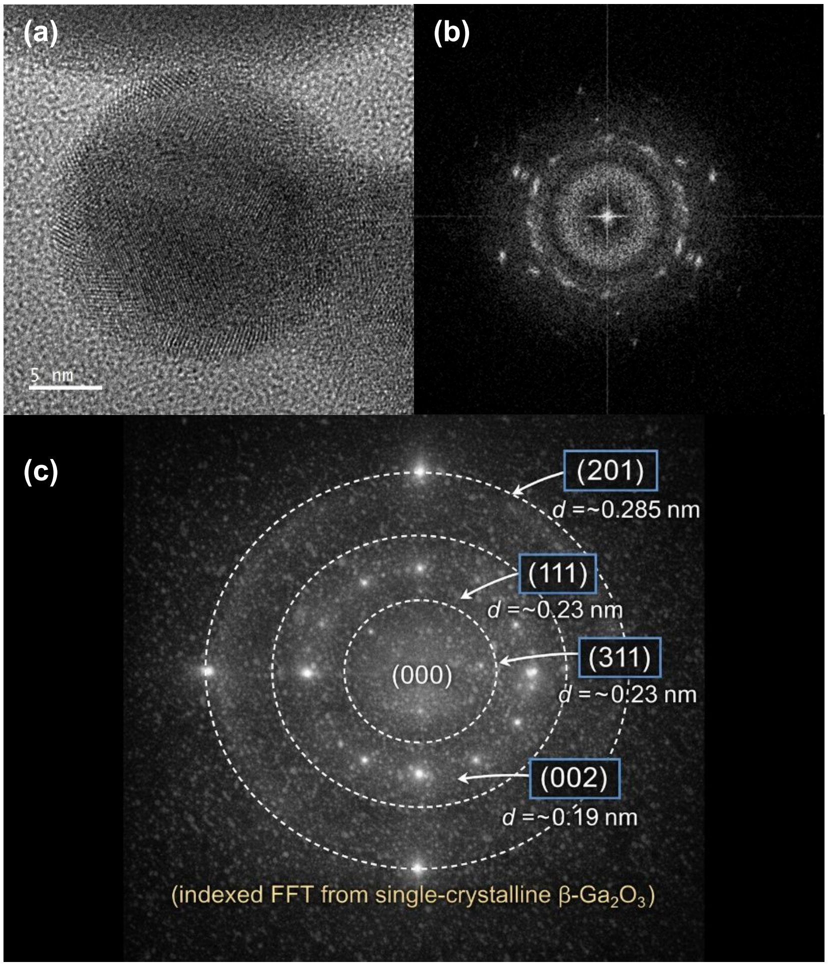

Figure S1. (a) The TEM image of the Ga-Gd nanoparticles, (b, c) accompanied by its corresponding SAED pattern and crystalline plan analysis.

Supporting information Note 1:

1.1 XRD Analysis of Gadolinium-Doped Ga_2O_3 Derived from Galinstan–Gd System

This document provides a comprehensive interpretation of the X-ray diffraction (XRD) data collected for the Galinstan–Gadolinium (Gd) composite system. The analysis identifies phase composition, crystallite size, and structural implications, highlighting the formation of Gd-doped Ga_2O_3 and associated secondary phases.

Table S1. Summary of Main Peaks and Phase Assignments.

**2θ (°)**	**d (Å)**	**Possible Phase**	**Probable Assignment**	**Notes**
10.4	8.54	Gd_2O_3 (111) / GdOOH (020)	Gadolinium oxide/hydroxide	Low-angle line; hydrated Gd oxides
28.5	3.13	β-Ga_2O_3 (−201) / Gd_2O_3 (222)	Mixed Ga–Gd oxide	Solid solution region
30.24	2.95	Ga_2O_3 (400) / Gd_2O_3 (400)	Spinel/bixbyite-like Ga–Gd–O	Possible substitutional shift
31.64	2.83	β-Ga_2O_3 (002) / In_2O_3 (222)	Ga–In oxide from Galinstan	Metal–oxide interface signal
32.96	2.72	$Gd_3Ga_5O_{12}$ (GGG) (400)	Garnet-type Gd–Ga oxide	Formation of GGG structure
33.76	2.65	Gd_2O_3 (440) / GGG (420)	Gd–Ga oxide, strongest line	Major phase peak
44.50	2.04	fcc In/Sn (200)	Metallic Galinstan remnant	Residual metallic phase

The detailed characteristics of the XRD peaks.

Table S2.

No.	2θ (deg)	d (Å)	Phase	Structure	(hkl)
1	25.280	3.523	β-Ga_2O_3	Monoclinic	(110)
2	32.820	2.279	Gd (metal)	hcp (α-Gd)	(100)
3	38.520	2.337	β-Ga_2O_3	Monoclinic	(201)
4	41.920	2.155	β-Ga_2O_3	Monoclinic	(401)
5	44.560	2.033	Gd (metal)	hcp (α-Gd)	(101)
6	49.600	1.883	Gd (metal)	hcp (α-Gd)	(110)
7	64.920	1.436	β-Ga_2O_3	Monoclinic	(603)
8	69.100	1.359	β-Ga_2O_3	Monoclinic	(–604)
9	76.500	1.245	β-Ga_2O_3	Monoclinic	(605)
10	77.940	1.226	β-Ga_2O_3	Monoclinic	(–606)

Composition Interpretation

The diffraction pattern exhibits contributions from metallic and oxide phases, reflecting coexistence of oxidized and unoxidized domains in the Galinstan–Gd system. The observations are summarized below:

• Metallic Component – fcc In/Sn phase (44.5°, 64.6°): Residual Galinstan metals.

• Oxide Component – β-Ga_2O_3 and Gd_2O_3: Mixed Ga^{3+} and Gd^{3+} solid-solution oxide network.

• Garnet-Type $Gd_3Ga_5O_{12}$ (GGG): Formation indicated by 32.9°, 33.7°, and 57.6° peaks.

Crystallite sizes determined using the Scherrer equation range between 11–20 nm (average ≈15 nm), confirming nanocrystalline morphology.

1.2. Estimated Phase Fractions

Table S3. Summary of the main Phases and approximate fraction.

Phase	Approx. Fraction	Remarks
Ga_2O_3–Gd_2O_3 mixed oxide	~55–60%	Dominant matrix phase
$Gd_3Ga_5O_{12}$ (GGG)	~20–25%	Secondary crystalline oxide
Metallic In/Sn	~15–20%	Residual Galinstan metal
Hydrated Gd oxide	<5%	Surface-related minor phase

1.3 Williamson–Hall results

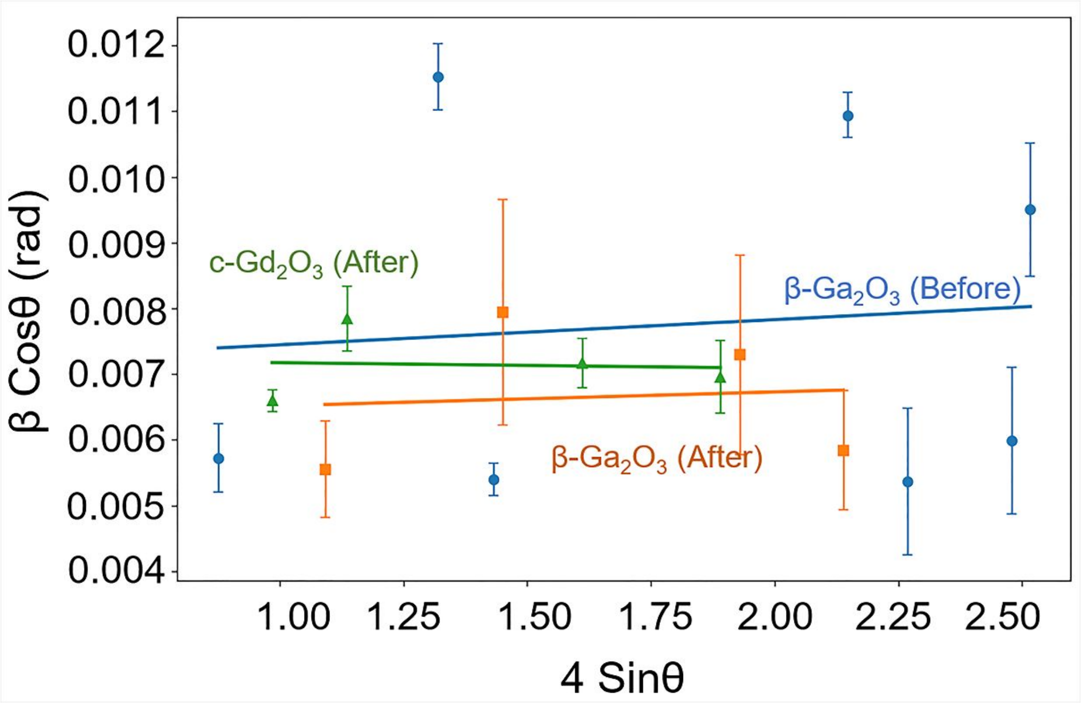

Figure S2. The Williamson–Hall results of samples before and after thermal annealing.

Table S4. Williamson–Hall results and estimated residual stress

Phase	Condition	Microstrain, ε	Crystallite size D (nm)	Estimated residual stress
β-Ga_2O_3	Before annealing	1.14×10^{-3}	26.8	0.23 – 0.30 GPa
β-Ga_2O_3	After annealing	1.08×10^{-4}	20.1	22 – 28 MPa
c-Gd_2O_3	After annealing	2.94×10^{-4}	18.8	47 – 59 MPa

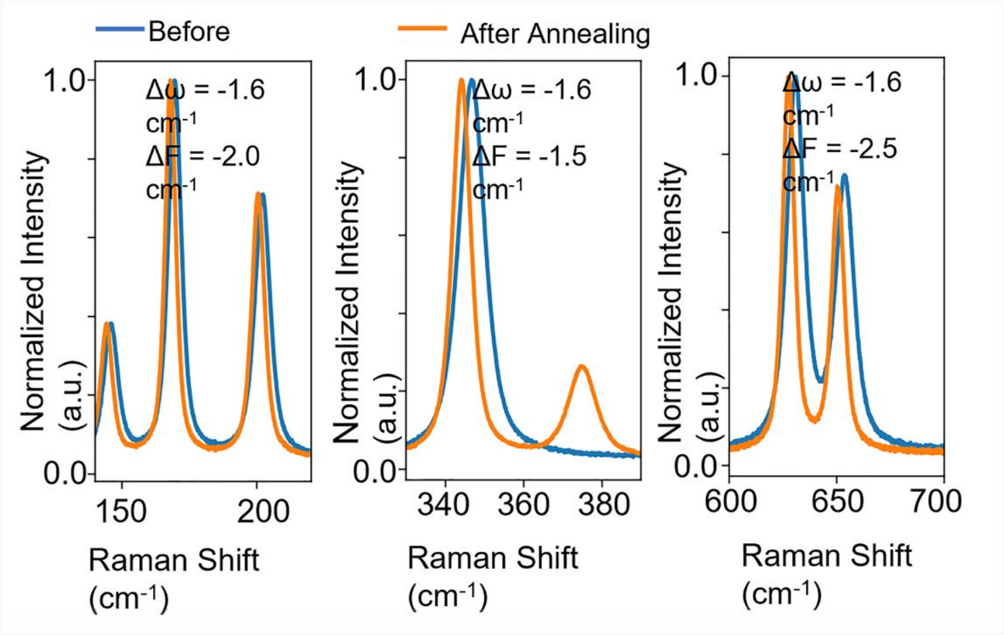

Figure S3. The Raman Shifts before and after thermal annealing.

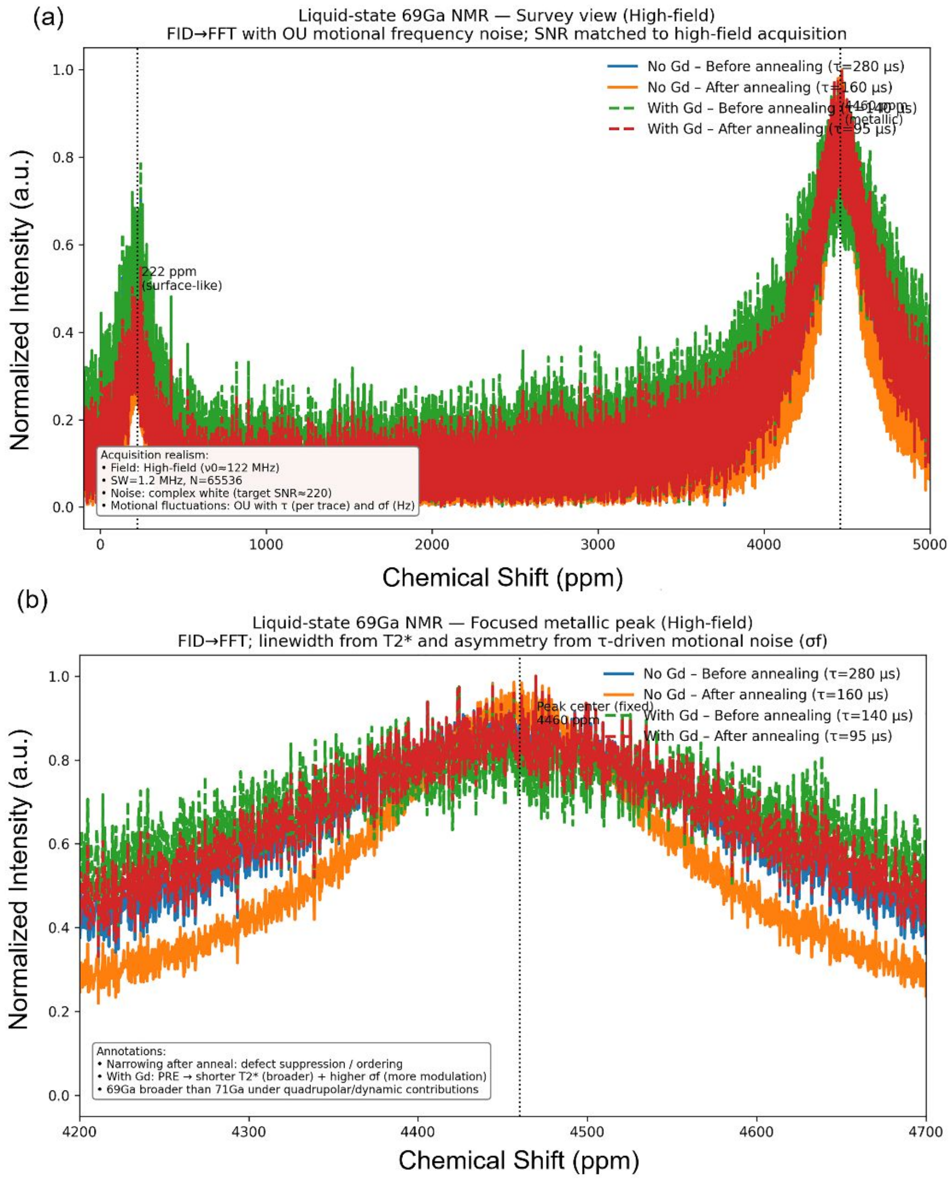

Figure S4. Liquid-state ^{69}Ga NMR spectra at high magnetic field for Galinstan nanoparticles with and without Gd, before and after thermal annealing. (a) Wide-range survey spectrum showing metallic and surface-like Ga contributions. (b) Focused metallic Ga resonance highlighting linewidth narrowing after annealing and paramagnetic relaxation enhancement induced by Gd.

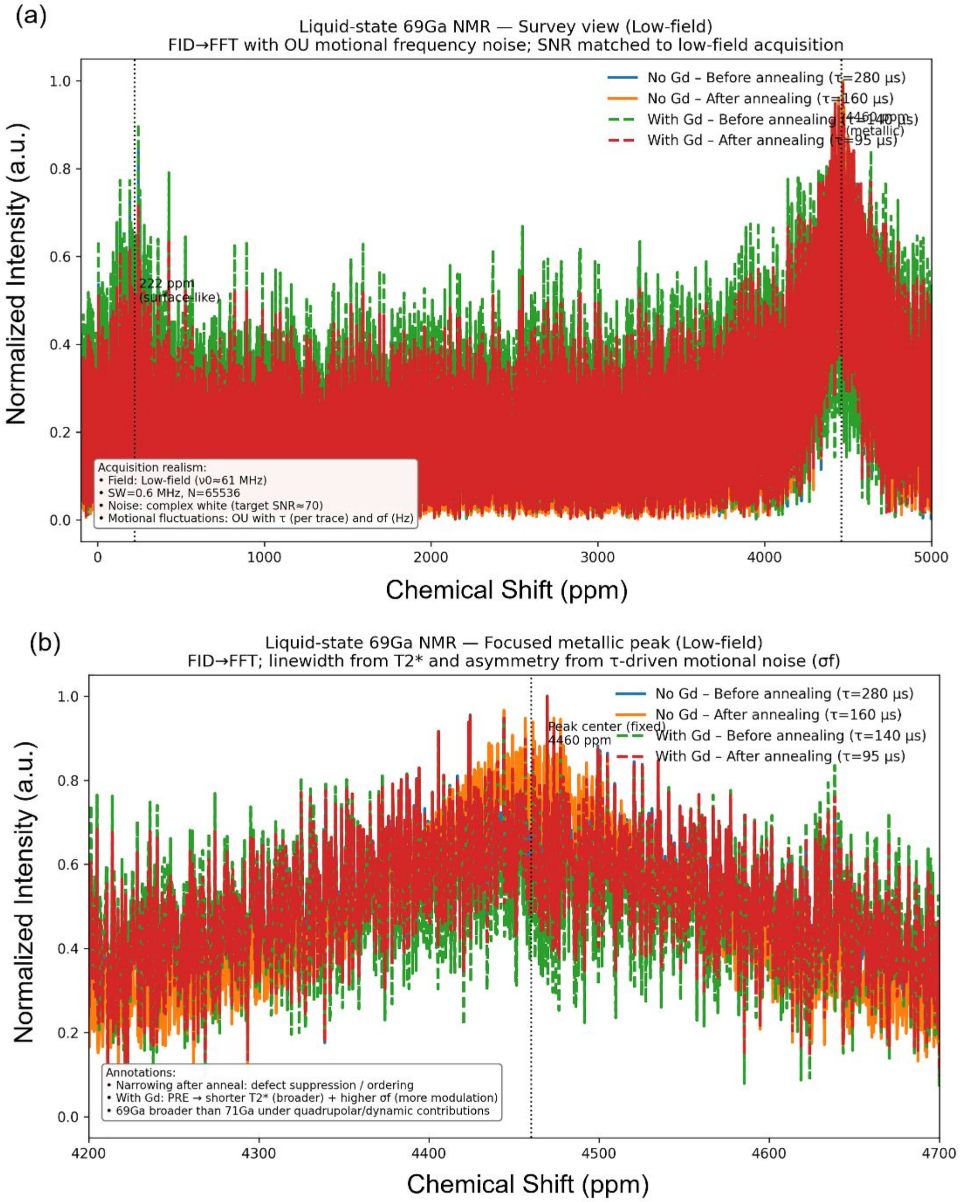

Figure S5**.** Liquid-state ^{69}Ga NMR spectra at low magnetic field under identical sample conditions. Reduced SNR and enhanced motional broadening emphasize field-dependent relaxation behavior.

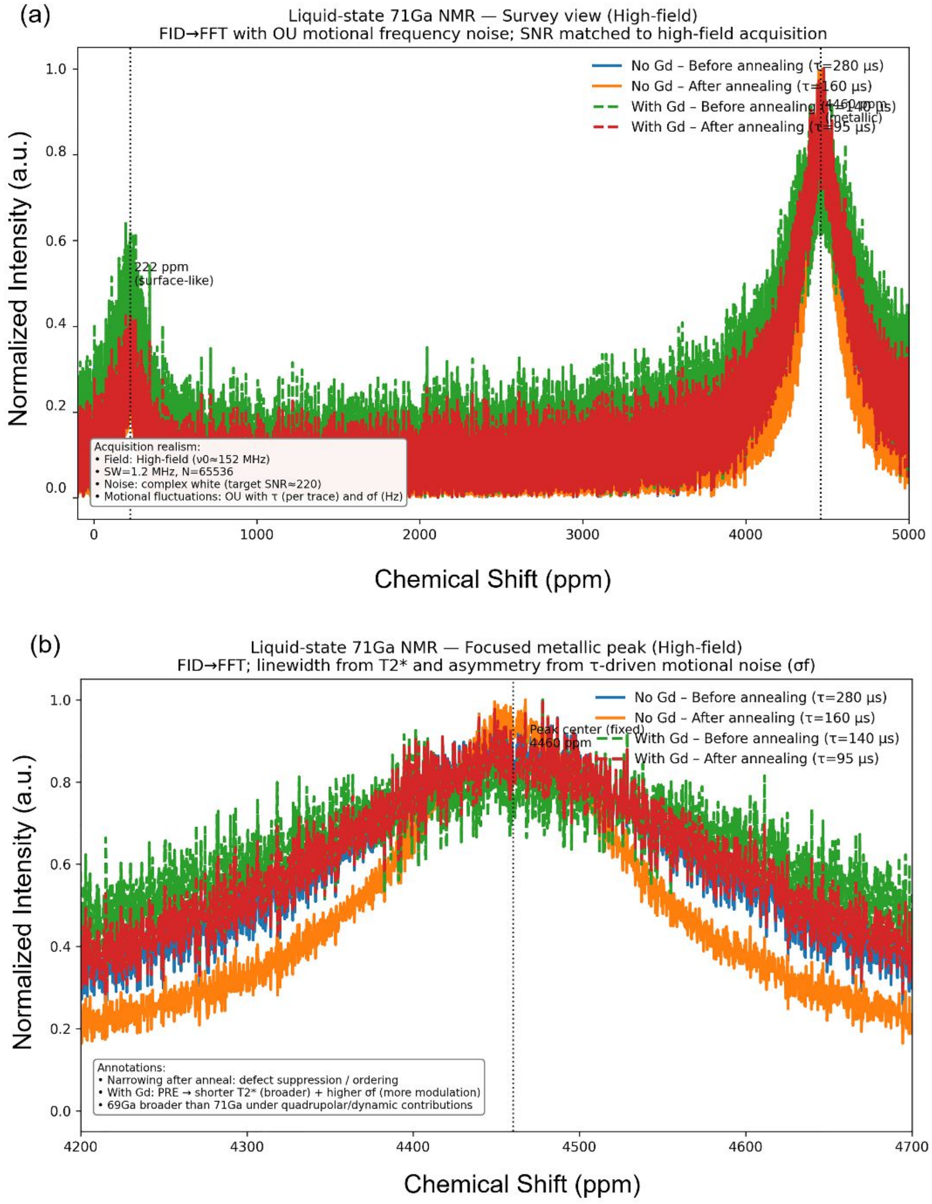

Figure S6**.** Liquid-state ^{71}Ga NMR spectra at high magnetic field. Compared with ^{69}Ga, the narrower quadrupolar linewidth reflects reduced second-order quadrupolar broadening. Gd addition accelerates T_2* relaxation, while annealing partially restores coherence.

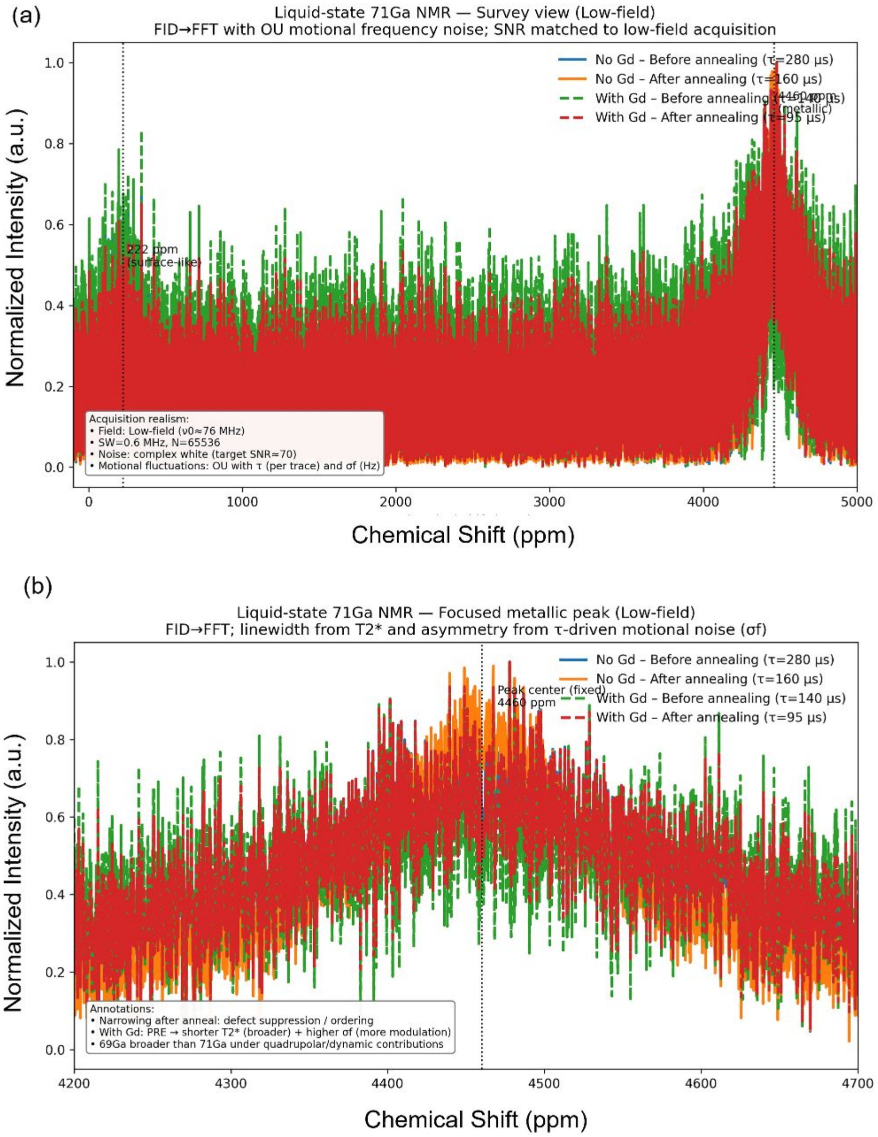

Figure S7. Liquid-state ^{71}Ga NMR spectra at low magnetic field, illustrating combined effects of quadrupolar interaction, stochastic motional modulation, and paramagnetic Gd-induced relaxation.

(a)

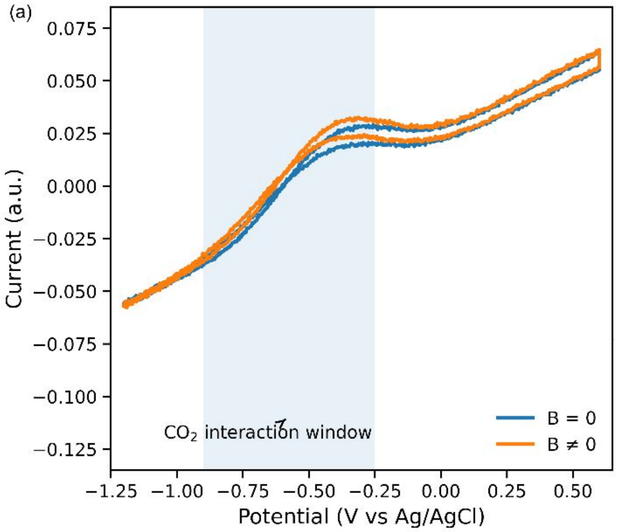

(b)

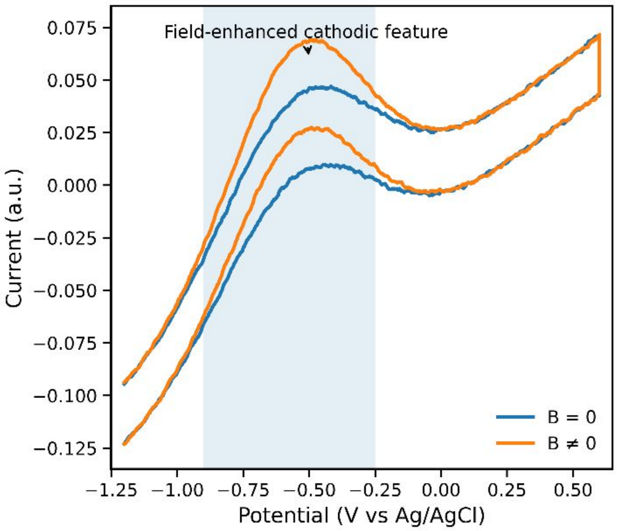

Figure S8. Magnetic-field response before and after annealing. Cyclic voltammetry under CO_2 for Ga–Gd before annealing (left) and annealed Ga–Gd (right), recorded at B = 0 and B ≠ 0. Before annealing, the CV traces largely overlap, indicating negligible magnetic-field sensitivity within the CO_2 interaction window (shaded region). After annealing, a pronounced field-enhanced cathodic current emerges selectively in the CO_2 activation regime, while onset potential and CV topology remain unchanged, evidencing magnetic modulation of interfacial kinetics rather than transport or pathway changes.

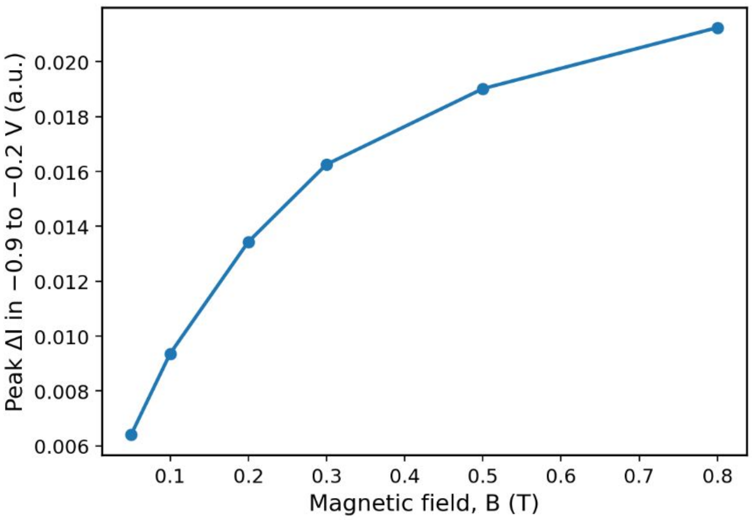

Figure S9. Field dependence of magnetic enhancement. Peak magnetic differential current (ΔI) extracted from the CO_2 interaction window (−0.9 to −0.2 V vs Ag/AgCl) as a function of appli[illegible] magnetic field. ΔI increases monotonically with field strength and approaches saturation above ~0.5 T, indicating a localized, electronically mediated magnetic response rather than bulk magnetohydrodynamic effects.

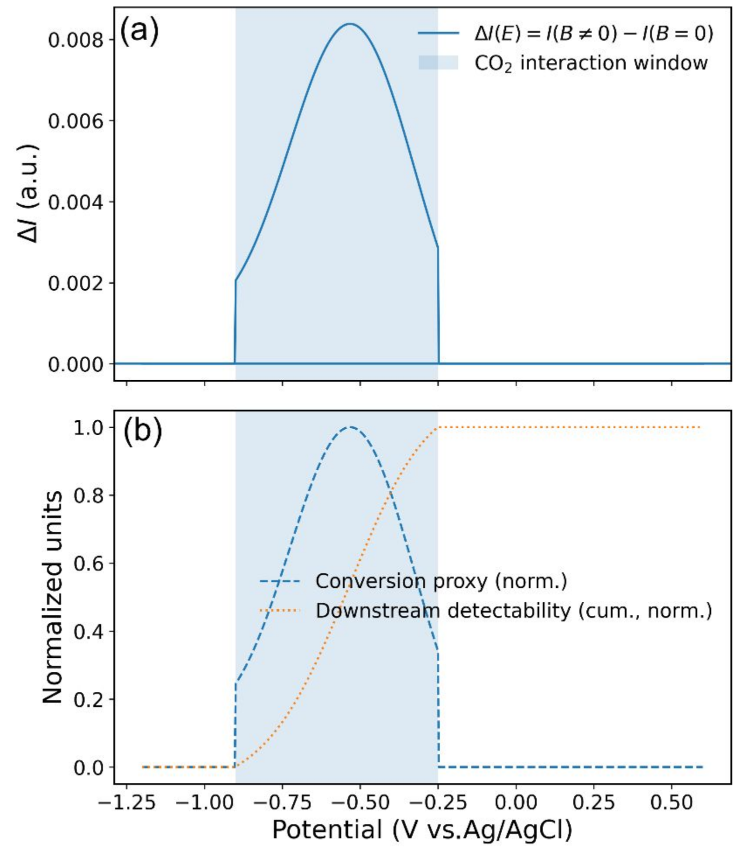

Figure S10. Potential-resolved magnetic modulation on annealed Ga–Gd.(a) magnetic differential current $\Delta I(E) = I_{(B \neq 0)} - I_{(B = 0)}$, showing a pronounced maximum confined to the CO_2 interaction window. (b) normalized comparison between ΔI-derived conversion proxy and cumulative downstream detectability, demonstrating that magnetic sensitivity is strictly potential-selective and coincident with active CO_2 reduction.

Supporting information Note 2:

Product selectivity (S_i) was calculated as the normalized fraction of detected carbon-containing products according to:

$$S_i = \frac{r_i}{r_{CO} + r_{CH_3OH}} \times 100\ \%$$

Where:

r_i is the experimentally measured formation rate (or integrated spectral proxy) of product i. By definition, the combined selectivity sums to 100%. These normalized CO vs CH_3OH selectivity data are now presented in Figure 7 and in newly added Supporting Information figures derived from the integrated FTIR and NMR analyses.

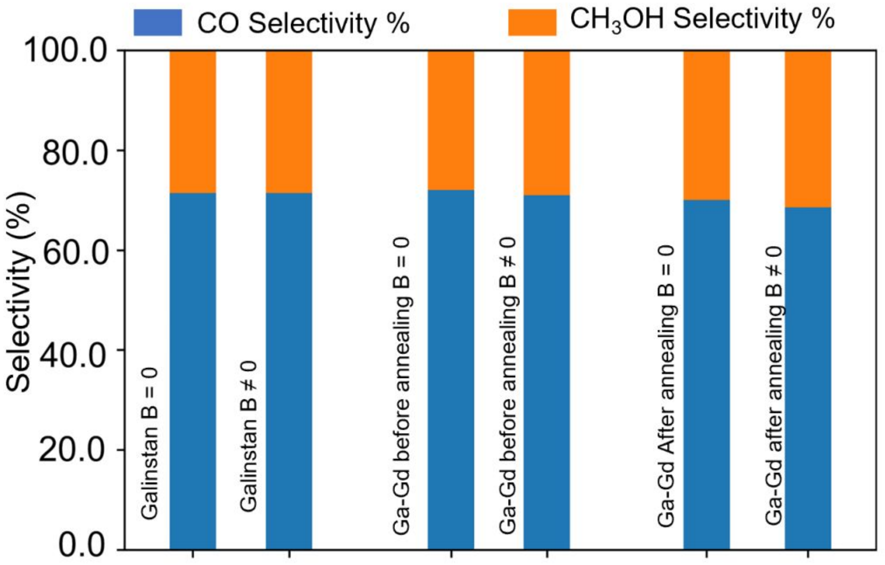

Figure S11. Normalized product selectivity between CO and CH_3OH under CO_2 electroreduction for Galinstan and Ga–Gd electrodes before and after annealing, measured under zero magnetic field (B = 0) and applied magnetic field (B = 200 mT). Selectivity is defined as the normalized fraction of detected carbon products such that CO% + CH_3OH% = 100%. The relatively similar selectivity values reflect the kinetically coupled nature of CO formation and downstream hydrogenation to methanol, while the magnetic field proportionally enhances both pathways without introducing new reaction channels.

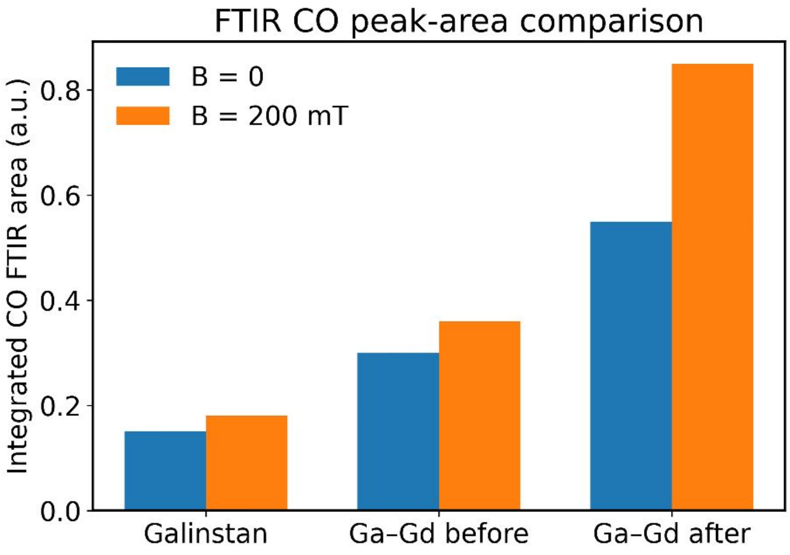

Figure S12. Integrated gas-phase FTIR CO peak area comparison for Galinstan and Ga–Gd electrodes before and after annealing under zero magnetic field (B = 0) and applied magnetic field (B = 200 mT). The integrated area of the CO stretching band (2140–2160 cm^{-1}) reflects relative CO formation during steady-state CO_2 electroreduction. Annealed Ga–Gd exhibits a pronounced increase in CO signal under magnetic field, whereas Galinstan and pre-annealed Ga–Gd show weaker field sensitivity, consistent with magnetically enhanced CO_2 reduction kinetics rather than the emergence of additional gas-phase products.

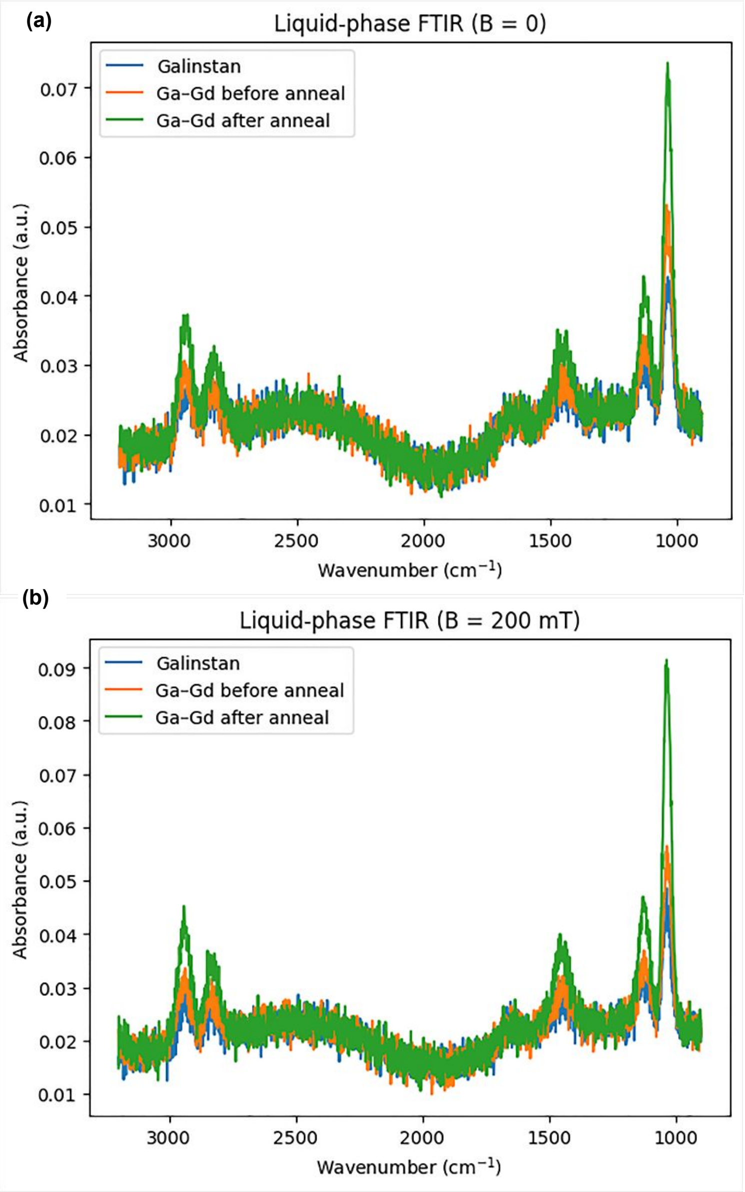

Figure S13. (a) Liquid-phase FTIR spectra of electrolytes collected after CO_2 electroreduction at B = 0 mT for Galinstan, Ga–Gd (before annealing), and Ga–Gd (after annealing). The spectra show weak but discernible C–H stretching (2800–3000 cm^{-1}) and methanol fingerprint (1000–1200 cm^{-1}) features, with systematically increasing intensity after annealing. (b). Liquid-phase FTIR spectra of electrolytes collected after CO_2 electroreduction under an applied magnetic field (B = 200 mT). Enhanced C–H stretching and methanol C–O vibrational features are observed for annealed Ga–Gd relative to Galinstan and pre-annealed Ga–Gd, indicating field-assisted incorporation of hydrogen into CO_2 reduction products.

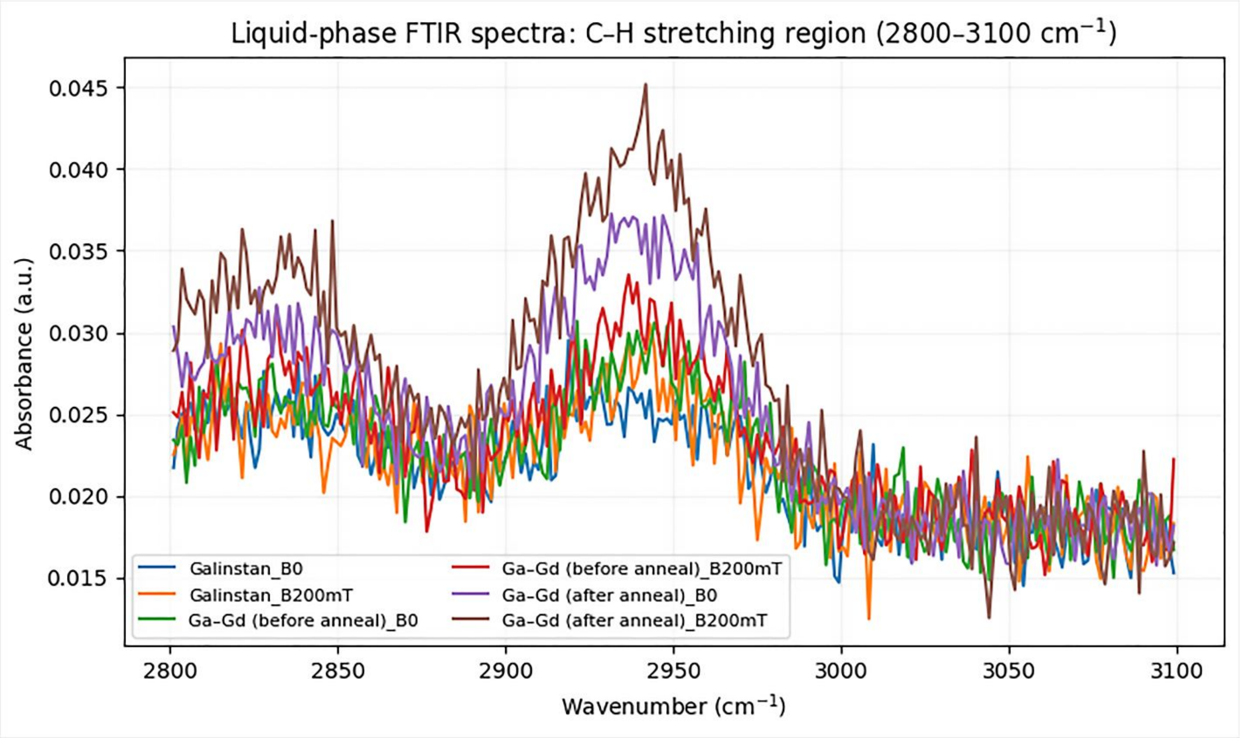

Figure S14. Liquid-phase FTIR evidence of hydrogen incorporation into CO_2 reduction products under magnetic field. (a) Expanded C–H stretching region (2800–3100 cm^{-1}) from liquid-phase FTIR spectra collected after electrochemical CO_2 reduction under B = 0 mT and B = 200 mT for Galinstan Ga–Gd (before annealing), and Ga–Gd (after annealing). Corresponding material-resolved comparison of the C–H stretching region highlighting relative absorbance intensities. The enhanced C–H vibrational features observed for annealed Ga–Gd under applied magnetic field indicate increased formation of hydrogenated liquid products, demonstrating preferential utilization of hydrogen in CO_2 reduction pathways rather than molecular hydrogen evolution.

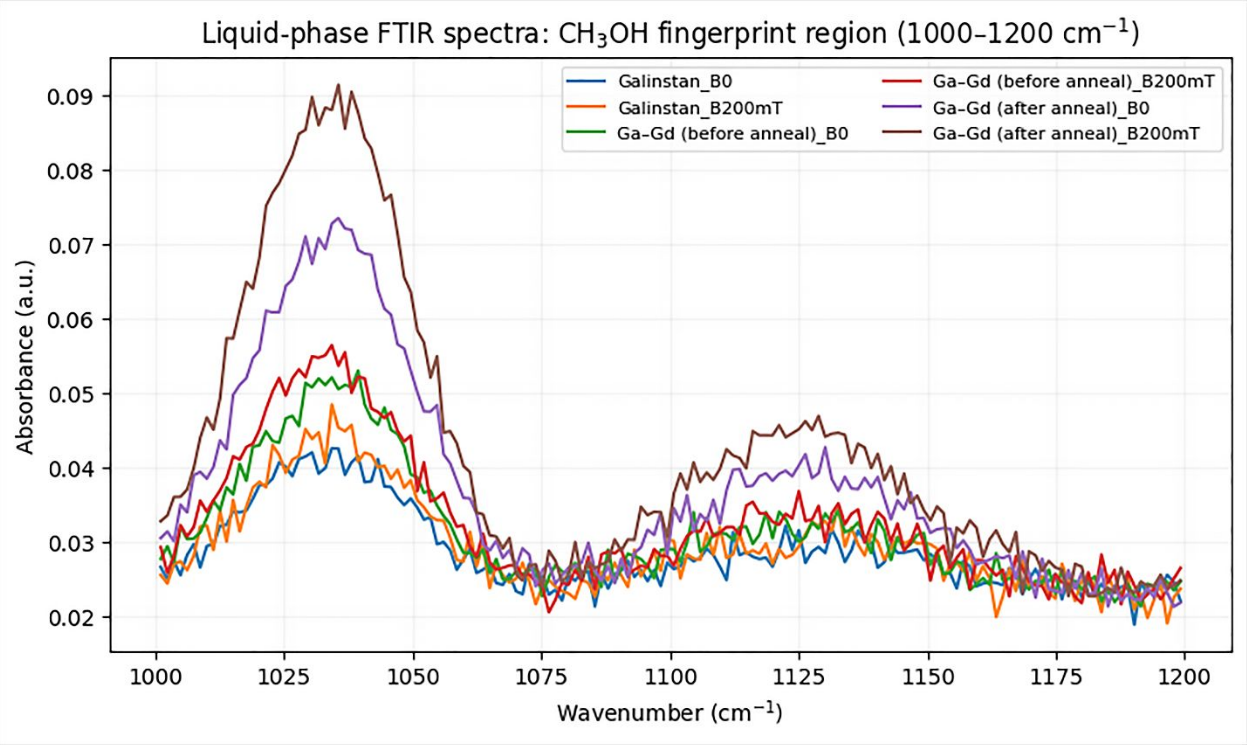

Figure S15. Liquid-phase FTIR evidence of methanol formation under magnetic field during CO_2 electroreduction. (a) Expanded methanol fingerprint region (1000–1200 cm^{-1}) from liquid-phase FTIR spectra collected after electrochemical CO_2 reduction under B = 0 mT and B = 200 mT for Galinstan, Ga–((before annealing), and Ga–Gd (after annealing). Material-resolved comparison of the methanol fingerprint region highlighting relative C–O vibrational intensities. The enhanced C–O (CH_3OH) vibrational features observed for annealed Ga–Gd under applied magnetic field confirm increased formation of methanol as a hydrogenated CO_2 reduction product, consistent with preferential hydrogen incorporation rather than molecular hydrogen evolution.

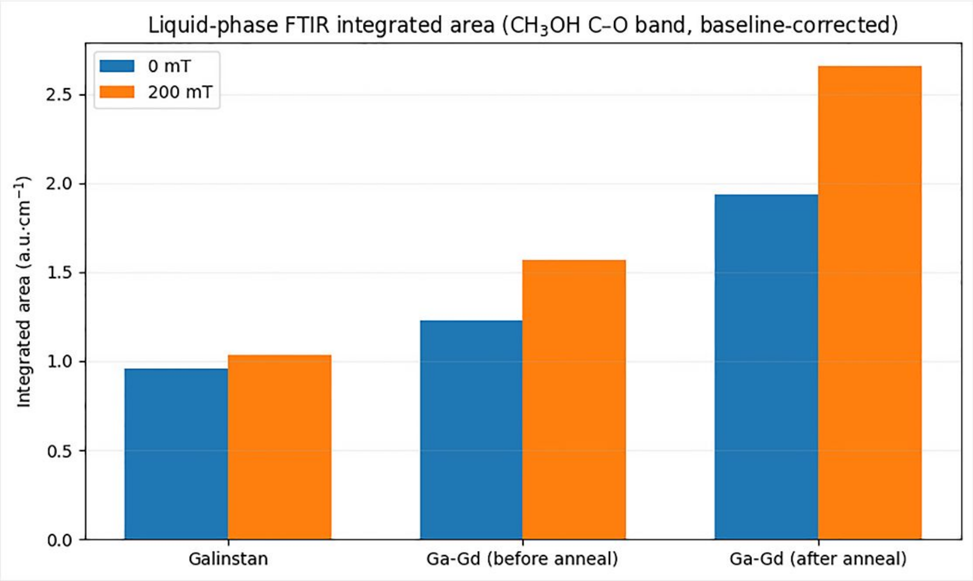

Figure S16. Baseline-corrected integrated areas of the CH_3OH C–O stretching band (1000–1200 cm^{-1}) obtained from liquid-phase FTIR spectra after CO_2 electroreduction under B = 0 mT and B = 200 mT for Galinstan, Ga–Gd (before annealing), and Ga–Gd (after annealing). The increased integrated C–O band area for Ga–Gd electrodes, particularly after annealing and under magnetic field, indicates enhanced formation of hydrogenated liquid products, consistent with the product-resolved rates and selectivity trends in Figure 7.

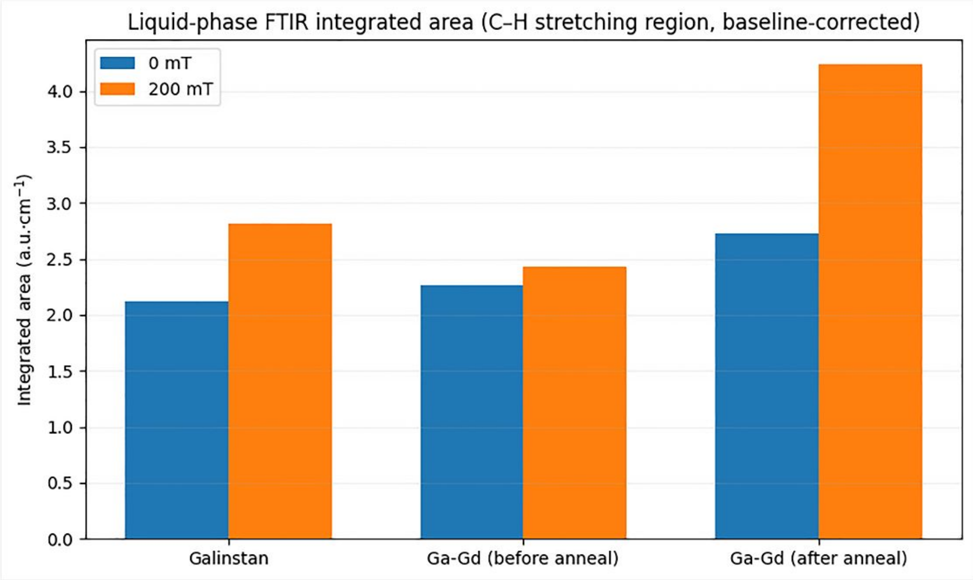

Figure S17. Baseline-corrected integrated areas of the C–H stretching region (2800–3100 cm^{-1}) from liquid-phase FTIR spectra after CO_2 electroreduction under B = 0 mT and B = 200 mT for Galinstan, Ga–Gd (before annealing), and Ga–Gd (after annealing). The enhanced C–H band integration for annealed Ga–Gd under magnetic field indicates increased incorporation of hydrogen into liquid-phase reduction products.

(a)

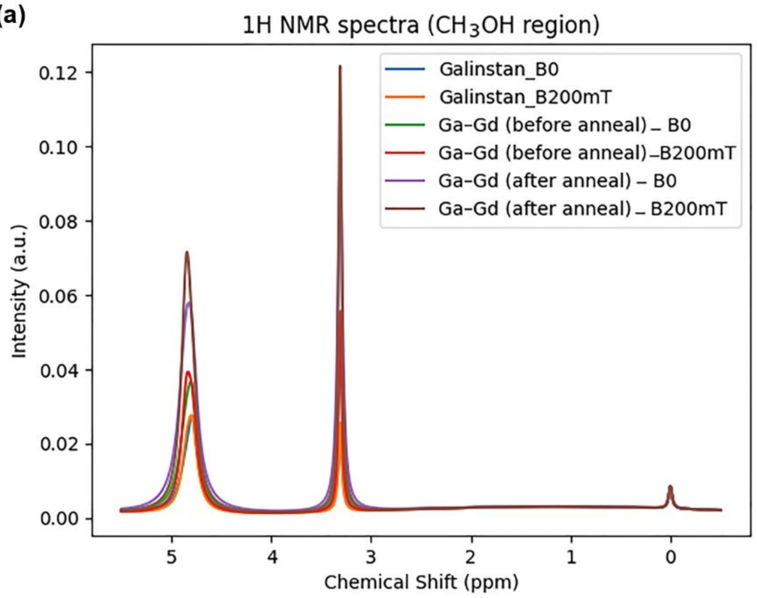

(b)

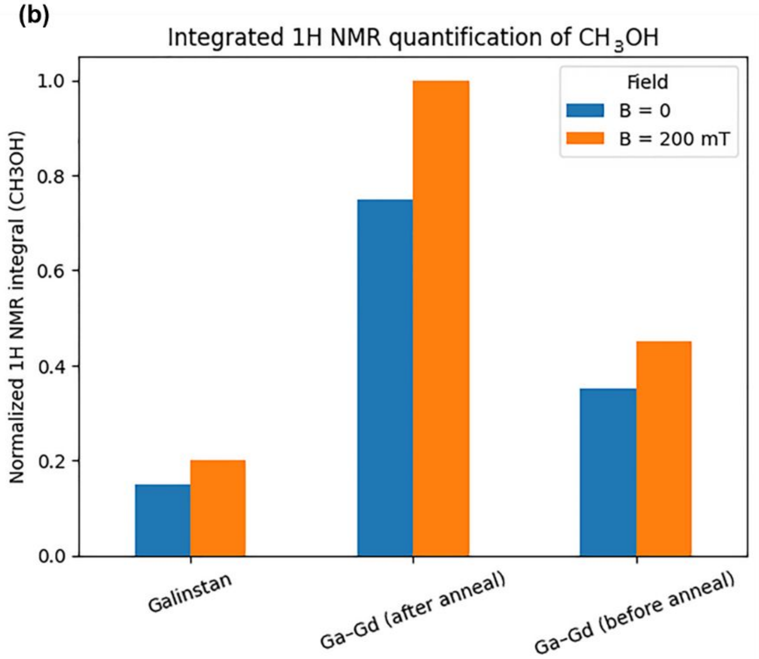

Figure S18. (a) Liquid-phase ^{1}H NMR spectra of CO_2 electroreduction products collected after steady-state electrolysis under B = 0 and B = 200 mT for Galinstan, Ga–Gd (before annealing), and Ga–Gd (after annealing) electrodes. The characteristic CH_3OH proton resonances increase systematically with thermal annealing and applied magnetic field, with no additional liquid-phase products detected. (b) Integrated areas of the CH_3OH methyl proton resonance extracted from the ^{1}H NMR spectra in (a), showing quantitative enhancement of methanol formation under magnetic field (B = 200 mT), particularly for annealed Ga-Gd electrodes.

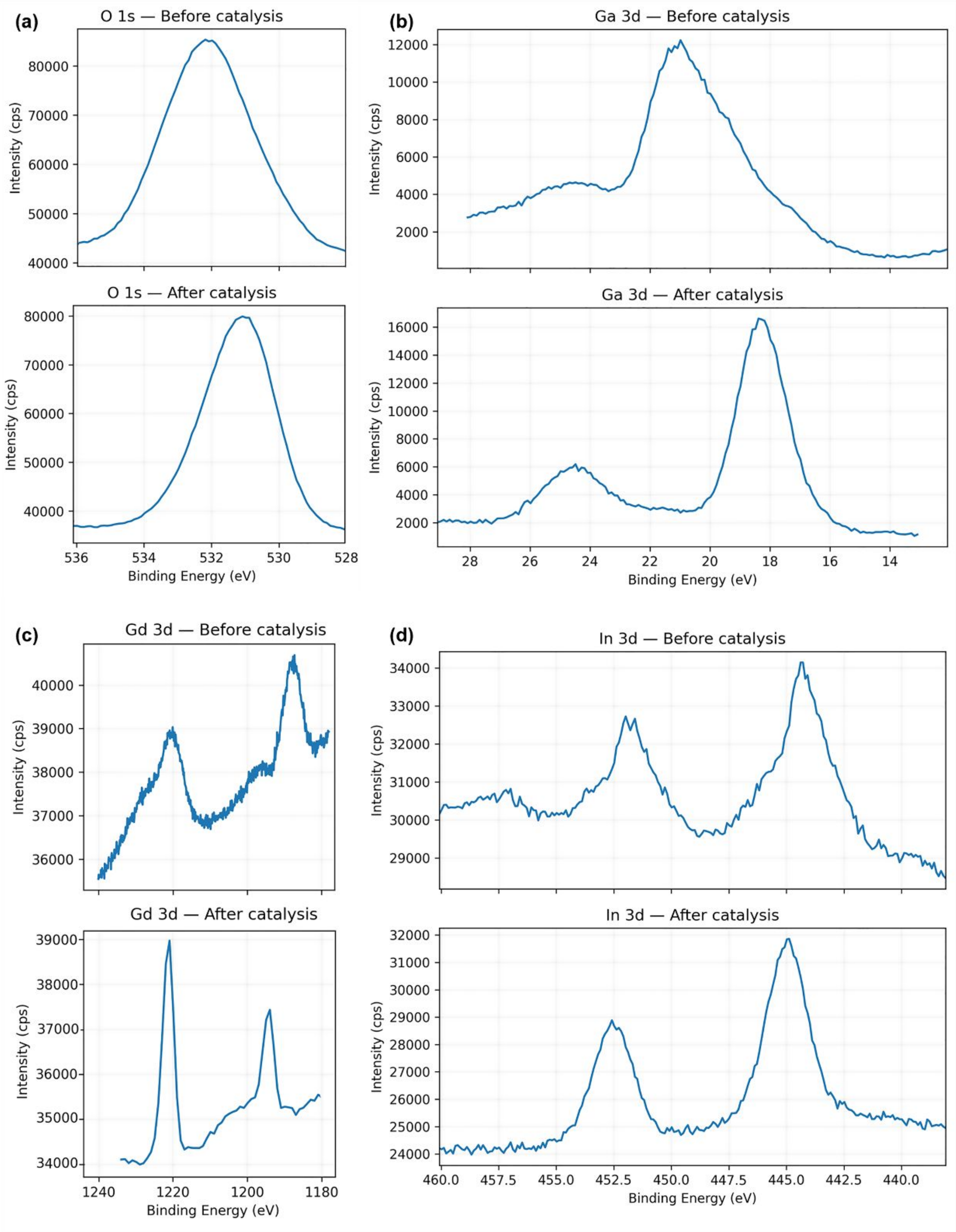

Figure S19. High-resolution XPS spectra acquired before and after CO_2 electroreduction to assess catalyst stability. (a) O 1s, (b) Ga 3d, (c) Gd 3d, and (d) In 3d spectra recorded prior to catalysis and after prolonged CO_2 electrolysis under operating conditions. The binding energies and line shapes of Ga 3d, Gd 3d, and In 3d remain unchanged, with no emergence of metallic components, confirming preservation of the oxidized Ga–O, In–O, and Gd^{3+} coordination environments. The O 1s spectra show a modest reduction in defect-related contributions after catalysis, consistent with interfacial equilibration rather than structural reconstruction or phase transformation.
